# Moderate Temperature Reduction Changes the High‐Light Acclimation Strategy of Lettuce Plants

**DOI:** 10.1111/ppl.70298

**Published:** 2025-06-02

**Authors:** Tapio Lempiäinen, Dorota Muth‐Pawlak, Julia P. Vainonen, Eevi Rintamäki, Mikko Tikkanen, Eva‐Mari Aro

**Affiliations:** ^1^ Molecular Plant Biology, Department of Life Technologies University of Turku Turku Finland

**Keywords:** enviromental acclimation, non‐photochoemical quenching, photoinhibition, photosystem II repair, protein phosphorylation

## Abstract

In nature, environmental conditions are constantly changing, requiring plants to have numerous regulatory mechanisms to keep light harvesting and metabolism in balance. Here, we show that high light (HL) induces a much stronger non‐photochemical quenching (NPQ) when lettuce plants are exposed to 1500 μmol photons m^−2^ s^−1^ for 4 h at 13°C (low temperature, LT) compared to 23°C (growth temperature, GT). GT/HL treatment induced NPQ to relax during 1 h in darkness. In contrast, LT/HL treatment induced an exceptionally high NPQ that only partially relaxed during 1 h in darkness at GT. Such a high sustained NPQ (sNPQ) cannot be explained by the canonical NPQ mechanism(s). Instead, sNPQ was associated with a transient increase in phosphorylation of minor LHCII antenna proteins, LHCB4.1/LHCB4.2 and partial disassembly of PSII‐LHCII complexes. This coincided with increased expression of the light‐harvesting‐like proteins SEP2 and ELIP1.2, the PSII assembly proteins HCF173 and LPA3, and accumulation of the pre‐D1 protein, indicating delayed PSII repair. These results lead us to propose that under LT/HL, the phosphorylation of LHCB4.1/LHCB4.2 initiates the disassembly of PSII‐LHCII supercomplexes, allowing accumulated SEP2 to bind to CP47, presumably leading to quenching of the inner PSII core antenna. The free CP43 core antenna, released from PSII at an early stage of repair, is proposed to be protected by accumulated LPA3. Apparently, the cascades of regulatory mechanisms are specific to each combination of environmental changes, depending on their concomitant effects on chloroplast redox balance and PSII repair rate, with induced PSII core antenna quenching contributing to sNPQ.

## 1 Introduction

Photosynthesis converts light energy into a chemical form that is used to assimilate carbon dioxide into carbohydrates. Two light‐driven reactions, catalysed by photosystem II (PSII) and photosystem I (PSI), together with the cytochrome b_6_f complex, form the major thylakoid protein complexes involved in linear electron transfer (LET) and the coupled proton transport to the thylakoid lumen. LET ultimately reduces ferredoxin (Fd) at the acceptor side of PSI by electrons extracted from water by PSII. Reduced Fd is used by ferredoxin NADP^+^ reductase to form NADPH, while ATP synthase uses the proton gradient to produce ATP from ADP and P_i_. NADPH and ATP generated by light reactions are used in stromal metabolism, where carbon assimilation in the Calvin‐Benson‐Bassham (CBB) cycle is the major sink.

Both photosystems have inner and external light‐harvesting antenna that collect excitation energy and transfer it to the reaction centre (RC) chlorophylls (Chl) to ensure their function even in low light conditions. The PSII reaction centre (RC) proteins D1 and D2 bind only small amounts of Chl, and two inner or core antenna proteins, CP43 and CP47, contain most of the Chl pigments bound in the PSII core complex (Su et al. 2017). In angiosperms, the PSII inner antenna is associated with an external antenna system composed of three minor LHCII antenna, LHCB4, LHCB5 and LHCB6, and several major LHCII antenna composed of the trimers of LHCB1, LHCB2 and LHCB3, forming LHCII‐PSII supercomplexes (sc). The major LHCII antenna is referred to as strongly, moderately, and loosely bound trimers (S‐LHCII, M‐LHCII and L‐LHCII, respectively) according to their binding strength to the PSII core complex (Caffarri et al. 2009). In PSI, the inner antenna pigments are bound to the same proteins, PSAA and PSAB that form the RC complex. The external LHCA antenna belt of PSI is composed of four monomeric LHCA proteins (Mazor et al. 2015). In addition, PSI can receive excitation energy from the LHCII antenna ‘lake’ shared with PSII, either by forming specific phosphorylation‐dependent LHCII‐PSI sc or through the LHCA belt (Grieco et al. 2015; Schiphorst et al. 2021).

While the efficiency of light harvesting and the transfer of excitation energy to RCs are largely independent of temperature, even a modest decrease in temperature slows all enzymatic reactions, including the CBB cycle and stromal metabolism, and thus reduces the strength of electron sinks in the stroma (Hüner et al. 2022). Therefore, the exposure of plants to low temperatures (LTs), even under moderate light conditions, requires active mechanisms to dissipate excess energy. The combination of decreasing temperature and increasing light intensity is particularly challenging for the photosynthetic machinery and easily leads to the accumulation of electrons in the LET, increasing the likelihood of harmful side reactions and the generation of reactive oxygen species (ROS). In order to balance light reactions and metabolism to avoid potential ROS‐induced hazards under over‐excitation conditions, plants must increase the capacity of stromal metabolism by up‐regulating the CBB cycle and alternative electron sinks, or dissipate the excess energy in the light‐harvesting antenna system or via RC quenching (Schöner and Heinrich Krause 1990; Hüner et al. 2016; Herrmann et al. 2019; Bag et al. 2023; Grebe et al. 2024).

The excess excitation energy collected by the LHCII antenna is dissipated by several partially overlapping non‐photochemical quenching (NPQ) mechanisms (Bassi and Dall'Osto 2021; Ruban and Saccon 2022). The fastest mechanisms in angiosperms involve the energy‐ and zeaxanthin‐dependent components of NPQ (qE and qZ, respectively), which are activated by lumen acidification (Holzwarth et al. 2009; Nilkens et al. 2010; Niyogi and Truong 2013). In qE, the low lumen pH rapidly induces the protonation of the PSBS protein, which then alters the function of the major LHCII antenna system, leading to the dissipation of excess energy as heat. Activation of the qZ begins shortly after induction of qE, and often both NPQ mechanisms (qE and qZ) continue in parallel under excess light conditions. The xanthophyll cycle catalyses the reversible interconversion of violaxanthin via antheraxanthin to zeaxanthin. Violaxanthin promotes light harvesting while its de‐epoxidation to zeaxanthin promotes NPQ.

Apart from the canonical NPQ mechanisms discussed above, several types of sustained NPQ (sNPQ) mechanisms, characterised by a delayed relaxation of NPQ compared to that of qE, have evolved in different species. The mechanisms of sNPQ are poorly understood, but, particularly in overwintering evergreens, they have been reported to be associated with zeaxanthin accumulation and the expression of the light‐harvesting‐like (LIL) proteins, but their specific function at the molecular level is poorly defined (Demmig‐Adams and Adams 2006; Zarter, Adams, Ebbert, Cuthbertson, et al. 2006; Zarter, Adams, Ebbert, Adamska, et al. 2006; Levin and Schuster 2023; Ye et al. 2024). More recently, the specific phosphorylation of LHCII proteins and energy spillover from PSII to PSI have been attributed to sNPQ mechanisms in conifers (Bag et al. 2020; Grebe et al. 2020), and at least one type of sNPQ is known to be activated by the lipocalin protein in Arabidopsis ( *Arabidopsis thaliana* ) LHCII (qH) (Malnoë et al. 2017; Bru et al. 2022).

Despite several NPQ and other photoprotective mechanisms, PSII is known to undergo a continuous cycle of damage and repair under all light conditions (Tyystjärvi and Aro 1996), and the inhibition of PSII photochemical activity can only be detected when the rate of damage exceeds the rate of repair (Aro et al. 1993). The photoinhibited PSII core complexes with damaged D1 protein (Savitch et al. 2002) are not photochemically active, but it has recently been reported that they are still able to quench the excitation energy non‐photochemically (qI_RC_) (Nawrocki et al. 2021). However, it remains unclear how qI_RC_ and the PSII repair cycle function as part of the network of NPQ mechanisms.

**FIGURE 1:**
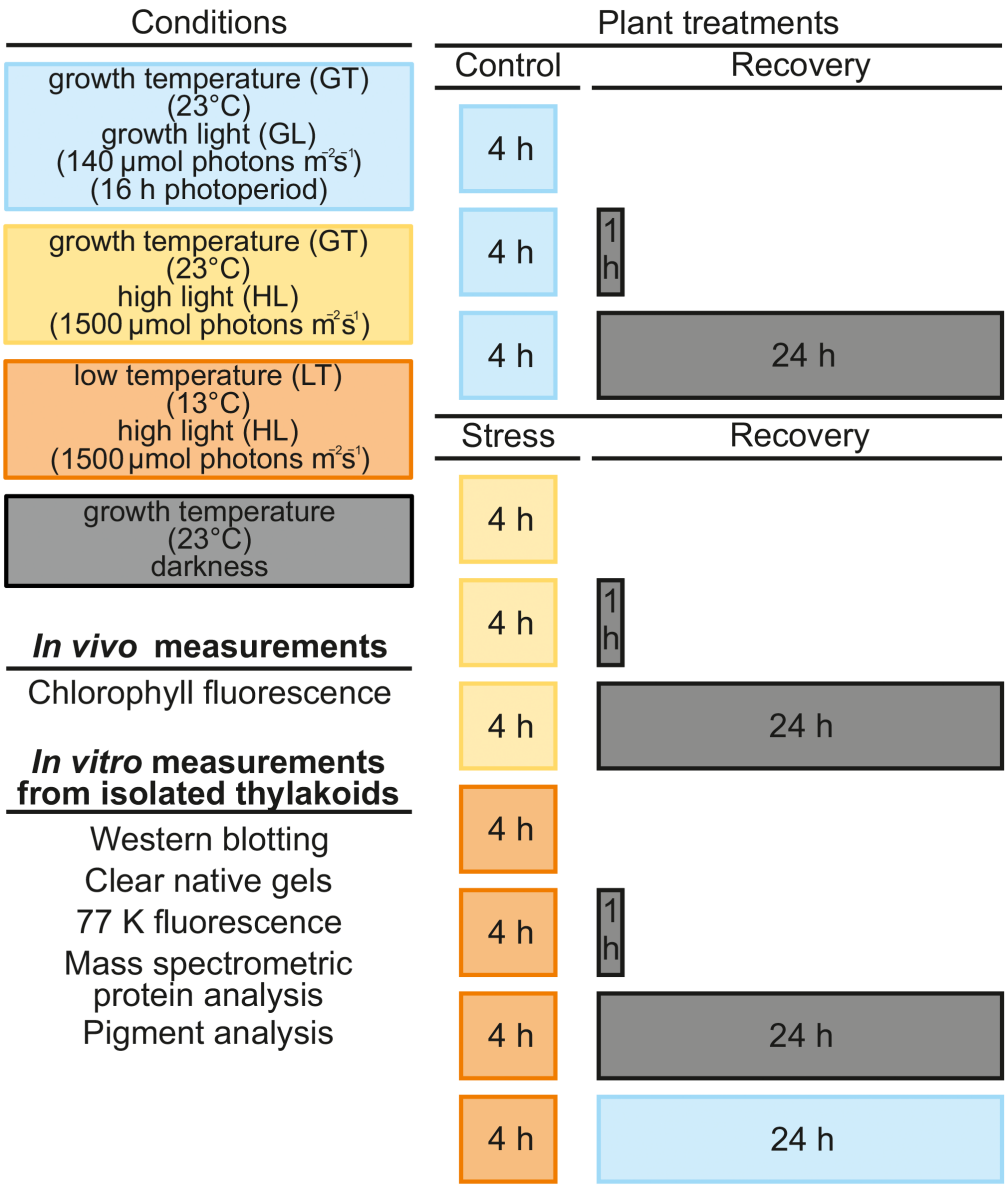
Experimental set‐up to study the short‐term acclimation mechanisms of the photosynthetic apparatus of lettuce to excessive excitation stress induced by simultaneous high light (HL) and low temperature (LT) stress. Long‐day grown lettuce plants were treated at 13°C under white light of 1500 μmol photons m^−2^ s^−1^ for 4 h (LT/HL) (orange boxes), while control plants were kept at growth temperature and light conditions (23°C, 140 μmol photons m^−2^ s^−1^) (GT/GL) (light blue boxes), or treated at HL in growth temperature (GT/HL) (yellow boxes), after which all plants were transferred to recover for 1 and 24 h in darkness (grey boxes) or for 24 h in normal long‐day conditions at GT/GL. In vivo Chl fluorescence measurements were performed on all samples. In addition, thylakoids were isolated from LT/HL‐treated plants and from GT/GL control plants directly after the treatment (blue and orange boxes) and subsequent recovery periods of 1 and 24 h in darkness or growth conditions. Isolated thylakoids were used for biochemical analyses and 77 K in vitro Chl fluorescence measurements.

Here, we investigated how a 10°C decrease in temperature affects the induction and relaxation of NPQ in lettuce. A decrease in temperature was found to induce a strong sNPQ that cannot be explained by the previously described NPQ mechanisms. To unravel the processes behind such a strong sNPQ in lettuce and to investigate the underlying processes behind the slow relaxation of sNPQ in the dark at physiological temperature, we used biochemical, biophysical and proteomic approaches according to the experimental protocol in Figure 1.

## 2 Materials and Methods

### 2.1 Lettuce Growth Conditions, Light Treatments and Recovery Conditions

Hilde White Boston cultivar of lettuce ( *Lactuca sativa* ) was grown in a 16 h‐photoperiod in moderate white light (140 μmol photons m^−2^ s^−1^) with POWERSTAR HQI‐T 400 W/D metal halide lamps (OSRAM GmbH) as the light source and at a growth temperature of 23°C (GT) (light blue boxes) (Figure 1). The plants used in the experiments were 4 weeks old.

Part of plants were treated with high light (HL, 1500 μmol photons m^−2^ s^−1^) for 4 h with Heliospectra Dyna LED lamps at LT (13°C) (orange boxes) (Figure 1), and the control plants were illuminated for 4 h at GL (light blue boxes). After the light treatment, one part of the plants from both sets was transferred to recover in darkness at GT for 1 h, and for 24 h (grey boxes), and the other part was transferred to recover in growth conditions (GT/GL) for 24 h (light blue boxes). These plants were subjected to a comprehensive analysis of PSII photoprotection mechanisms. In addition, lettuce plants were also exposed to a HL treatment alone (yellow box) (GT/HL), but were only monitored by Chl fluorescence after HL treatment as, under these conditions, the induced sNPQ largely dissipated within 1 h in the dark.

### 2.2 Chl Fluorescence and P_M_ Measurements

Chl fluorescence was measured with a PAR‐FluorPen FP 110 using the saturating pulse method (Photon Systems Instruments) with default settings. The quantum yield of PSII photochemistry (Y(II)) was estimated as (*F*_*M*_′−*F*_0_′)/*F*_*M*_′ in light‐ and as (*F*_*M*_−*F*_0_)/*F*_*M*_ in dark‐acclimated plants. NPQ was calculated as (*F*_*M*_^ref^/*F*_*M*_′)−1 in light‐ and as (*F*_*M*_^ref^/*F*_*M*_)−1 in dark‐acclimated plants, using the average of *F*_*M*_ from control plants transferred to darkness for 24 h as the reference value (*F*_*M*_^ref^). The effect of 4 h LT/HL treatment on maximal PSI oxidation (P_M_) was measured from treated and control plants after 1 h dark acclimation with Dual KLAS‐NIR (Heinz Walz GmbH) using the NIR MAX script (Schreiber and Klughammer 2016).

### 2.3 Thylakoid Isolation

Thylakoids were isolated from GT/GL and LT/HL‐treated and recovered plants similarly to as described in (Gunell et al. 2023) with minor modifications (see File S1). Chl concentration was determined in buffered acetone according to Porra et al. (1989).

### 2.4 Western Blotting

Western blot analysis was performed as described in Gunell et al. (2023) with minor modifications (see File S1).

### 2.5 Clear Native and 2D Gel Electrophoresis and Phosphoprotein Staining

Clear native gel electrophoresis (CN) and 2D gel electrophoresis were performed as described in Järvi et al. (2011) with minor modifications (see File S1). Phosphoprotein staining with ProQ was performed according to the manufacturer's instructions (Invitrogen), and the gels were imaged on a Perkin Elmer Geliance 1000 with a Cy3 filter.

### 2.6 77 K Chl Fluorescence

77 K Chl fluorescence measurements were performed with small thylakoid aliquots (50 μL) diluted to a Chl concentration of 10 μg/mL, to minimise self‐absorption. Thylakoids were excited with 480 nm light in liquid nitrogen and fluorescence was detected using an Ocean Optics S2000 spectrophotometer. Spectra were normalised to the 685 nm peak, and the ratio between the height of the 735 nm peak to the 685 nm peak (F735/F685) was calculated to illustrate the distribution of excitation energy between PSII and PSI.

### 2.7 Proteomics Analysis

Proteins from isolated thylakoids were solubilised, digested and desalted according to a previously described protocol (Huokko et al. 2019), with minor modifications (see File S1). The nLC‐ESI‐FAIMS‐MS/MS analyses were performed on a nanoflow HPLC system (Easy‐nLC 2000, Thermo Fisher Scientific) coupled to an Orbitrap Fusion Lumos mass spectrometer (Thermo Fisher Scientific) equipped with a high‐field asymmetric waveform ion mobility spectrometry (FAIMS Pro) interface in data‐independent acquisition (DIA) mode (see File S1).

Data analysis was performed using Spectronaut (version 16.1) software (Bruderer et al. 2015) (Biognosys). DIA data were searched against a customised FASTA file containing 37,834 protein sequences (File S5) using the Pulsar directDIA algorithm. The customised FASTA file contained the manually curated lettuce proteomes from UniProt (retrieved 2023.02.15) and Swiss‐Prot (retrieved 2023.03.28) (File S2). UniProt homologues of Swiss‐Prot sequences were manually removed from the combined database based on BLAST searches (Files S3 and S4). Peptide identification was performed with trypsin as an enzyme, allowing a maximum of two missed cleavages, as well as carbamidomethylation set as a static modification, while methionine oxidation, N‐terminal acetylation and lysine acetylation in addition to serine, threonine, and tyrosine phosphorylation were set as dynamic modifications. The FDR identification threshold (*q*‐value) for peptides and proteins was set at 0.01. Data for target proteins (identified based on homology to Arabidopsis sequences; Files S6 and S7) were extracted and analysed in Excel files. Protein abundances were normalised within samples to the average of the detected PSI, ATP synthase and Cyt b_6_f subunits to avoid, as much as possible, the effect of contaminants from the isolations and differential attachment of stromal proteins to the thylakoids under changing experimental conditions (e.g., a switch to HL rapidly recruits ribosomes to the thylakoid membrane), similarly to previous studies on the plant thylakoid proteome (Flannery, Pastorelli, et al. 2021; Flannery, Hepworth, et al. 2021). The original data and protein quantification project (Spectronaut file) are deposited in the PRIDE Archive database (Vizcaíno et al. 2016) (PXD055190).

### 2.8 Pigment Analysis

Pigments were extracted and separated according to Gilmore and Yamamoto (1991), with minor modifications (see File S1). Separated pigments were identified by their absorbance spectra and relative retention times. Relative pigment abundances were estimated by the area of chromatographic peaks detected at 440 nm and normalised within the sample to the area of Chl *a*.

## 3 Results

### 3.1 Temperature Reduction From 23°C to 13°C During HL Treatment of Lettuce Induces Strong sNPQ

**FIGURE 2:**
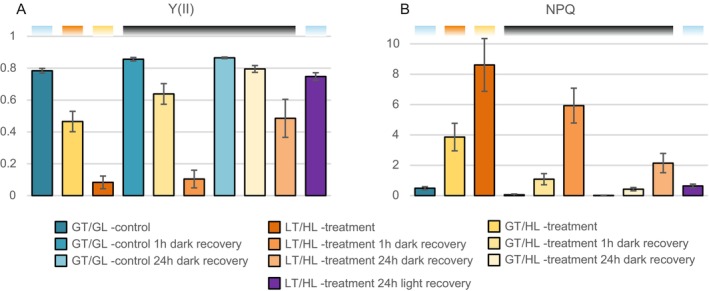
Comparison of low temperature and high light treatment (LT/HL) with high light treatment alone (GT/HL) on the formation of sustained excitation energy quenching in lettuce. (A) PSII quantum yield (Y(II)). (B) Non‐photochemical quenching (NPQ). Long day‐grown lettuce plants were illuminated under 1500 μmol photons m^−2^ s^−1^ of white light at 13°C for 4 h (LT/HL) or at 23°C (GT/HL). Control plants were kept at growth conditions (23°C, 140 μmol photons m^−2^ s^−1^) (GT/GL), after which all plants were transferred to recover at 23°C for 1 and 24 h in the dark or for 24 h in the long day growth conditions. *F*_0_′, *F*_0_, *F*_*M*_′, and *F*_*M*_ were determined with Fluorpen directly after the treatment and after subsequent recovery periods of 1 and 24 h. Y(II) was calculated as (*F*_*M*_′−*F*_0_′)/*F*_*M*_′ in light‐ and as (*F*_*M*_‐*F*_0_)/*F*_*M*_ in dark‐acclimated plants. NPQ was calculated as (*F*_*M*_^ref^/*F*_*M*_′)−1 in light‐ and as (*F*_*M*_^ref^/*F*_*M*_)−1 in dark‐acclimated plants, using as the reference value (*F*_*M*_^ref^) the average of *F*_*M*_ from control plants shifted to darkness for 24 h. Error bars show standard deviations among biological replicates (*n* = 4–16). Coloured bars above the graphs represent the light conditions in which the measurement was conducted: Light blue for growth light, orange and yellow for high light, and black for darkness.

Lettuce plants were exposed to high light at low temperature (LT/HL) and at growth temperature (GT/HL) for 4 h to investigate how photosynthetic acclimation differs when metabolism can operate at optimal temperature compared to limiting metabolism by lowering temperature by 10°C. Chl fluorescence analysis revealed a drastic decrease in PSII quantum yield (Y(II)) from 0.78 in GT/GL controls to 0.08 during the 4 h LT/HL treatment, but only a modest decrease to 0.45 during the 4 h GT/HL treatment (Figure 2A). After 1 h recovery in the dark at 23°C, Y(II) in GT/HL‐treated lettuce recovered to 0.65, but only to 0.10 in LT/HL‐treated lettuce (Figure 2A). After 24 h recovery in darkness at 23°C, Y(II) was fully recovered in GT/HL‐treated lettuce, but only to 0.49 in the case of LT/HL‐treated lettuce (Figure 2A), suggesting that part of the reduction in Y(II) was due to PSII inhibition, which did not recover in darkness. This interpretation was supported by the fact that Y(II) of LT/HL‐treated lettuce recovered to 0.75 within 24 h under GT/GL (Figure 2A), where the PSII repair cycle is active.

We then calculated the NPQ parameter in the control and treated lettuce (GT/HL and LT/HL, respectively) (Figure 2B), using the average *F*_*M*_ of dark recovered controls (GT/GL 24 h dark recovery) as a reference value. NPQ was normal at 3.86 in GT/HL‐treated lettuce and very high at 8.16 in LT/HL‐treated lettuce immediately after the treatments (Figure 2B). In GT/HL‐treated lettuce, the NPQ was mostly relaxed after 1 and 24 h recovery in darkness (Figure 2B). In contrast, the high NPQ in LT/HL‐treated lettuce relaxed slowly and was 5.93 after 1 h and 2.09 after 24 h recovery in darkness (Figure 2B). Since the comparison of fluorescence parameters between the LT/HL‐treated and GT/HL‐treated lettuce revealed typical NPQ in GT/HL‐treated lettuce but a high sNPQ in LT/HL‐treated lettuce (Figure 2B), we will from now on focus on elucidating the sNPQ mechanism(s) induced by the LT/HL treatment and compare the results with GT/GL controls.

Since LT is known to induce also PSI photoinhibition in some plant species (Sonoike 1996), we examined the effect of the 4 h LT/HL treatment on maximum PSI oxidation (P_M_) in comparison to the GT/GL control plants. The LT/HL treatment of 4 h had only a minor effect on PSI, as P_M_ was reduced from 1.75 in GT/GL control plants to 1.65 in 4 h LT/HL‐treated plants (Figure S1), indicating that the 4 h LT/HL treatment had only a minor effect on PSI function.

### 3.2 LT/HL Treatment and Recovery Alter Thylakoid Protein Phosphorylation and Composition of Pigment‐Protein Complexes

**FIGURE 3:**
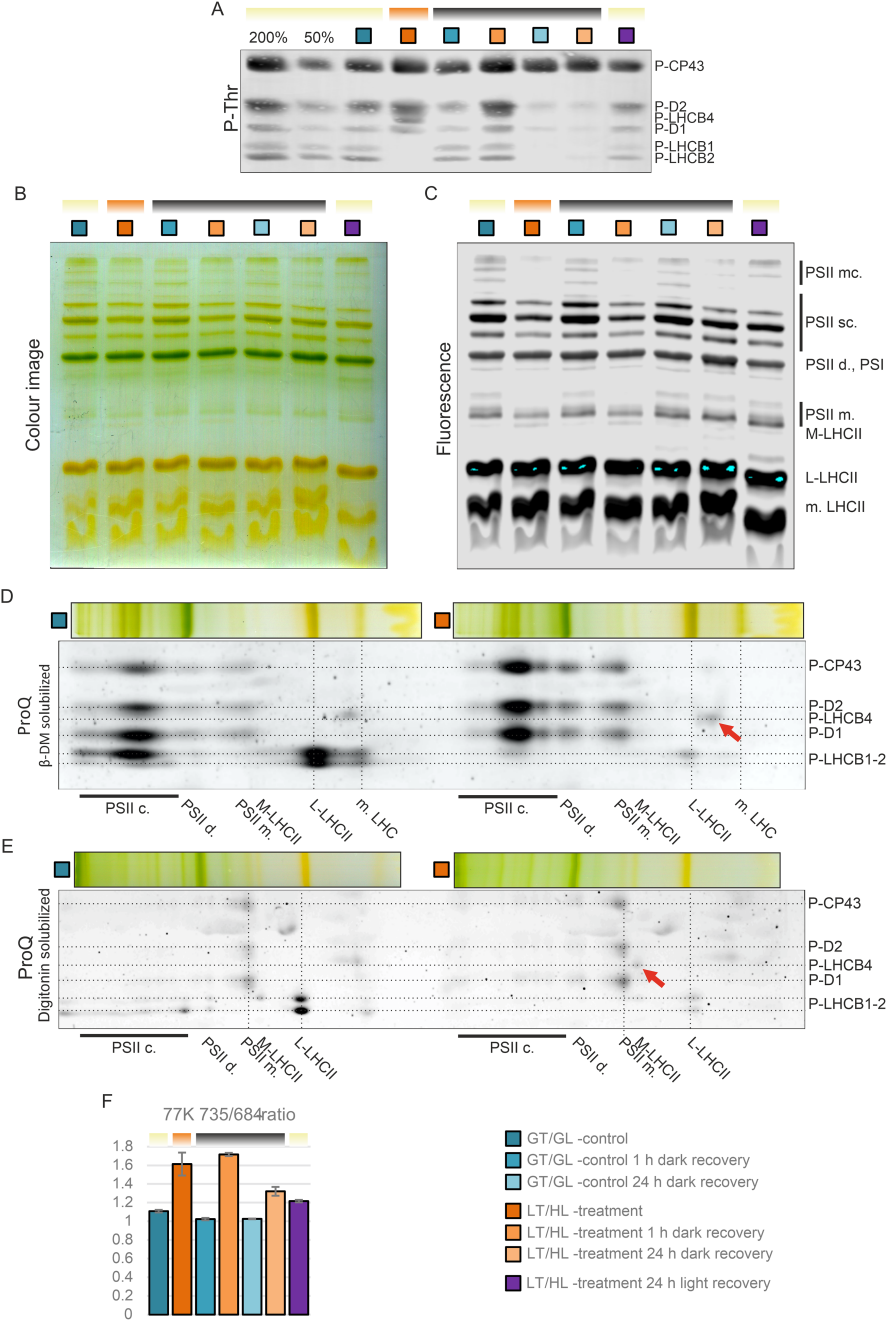
Effect of the low temperature and high light treatment, and the subsequent recovery, on phosphorylation‐dependent regulation of light harvesting in lettuce. (A) Immunodetection of protein phosphorylation. Thylakoid proteins were separated with SDS‐PAGE, transferred to the PVDF membrane and immunodetected with p‐Thr antibody and IR‐dye labelled secondary antibody. (B) Colour image and (C) Fluorescence image of β‐DM solubilised pigment‐protein complexes separated with clear native gel electrophoresis. The fluorescence of separated pigment‐protein complexes was visualised by exciting the complexes with a 685 nm laser with Odyssey CLx imager (light blue regions in the L‐LHCII indicate oversaturation). (D, E) Phosphoprotein staining (ProQ) of the 2D gels of thylakoid proteins solubilised with β‐DM (D) and digitonin (E) before electrophoresis in the first dimension. LCHB4 is indicated with red arrows. (F) Fluorescence ratio of PSI (735 nm) to PSII (685 nm) from the 77 K Chl fluorescence emission spectra of isolated thylakoids (F735/F685). Error bars show standard deviations among technical replicates (*n* = 3). Long day‐grown lettuce plants were illuminated under 1500 μmol photons m^−2^ s^−1^ of white light at 13°C for 4 h (LT/HL), while control plants were kept at growth conditions (23°C and 140 μmol photons m^−2^ s^−1^ with 16 h photoperiod) (GT/GL), after which all plants were transferred to recover for 1 and 24 h in darkness or for 24 h in long day growth conditions. Thylakoid membranes used in the analyses were isolated directly after the treatment and the recovery periods. Coloured bars above the graphs represent the temperature and light conditions of plants from which the thylakoids used in the analysis were isolated: Light blue for GT/GL, orange for LT/HL and black for GT/darkness. L‐LHCII, loosely bound LHCII trimer; m LHC, monomeric LHCII; M‐LHCII, moderately bound LHCII trimer; PSI, Photosystem I; PSII d, PSII dimer; PSII m, PSII monomer; PSII sc, PSII supercomplexes.

The phosphorylation of the LHCII proteins has previously been linked to sNPQ (Grebe et al. 2020), which prompted us to analyse the phosphorylation of thylakoid proteins with the p‐Thr antibody from the GT/GL control, LT/HL‐treated and recovered lettuce plants (Figure 3A). The LT/HL treatment increased the phosphorylation level of the PSII core proteins CP43, D2 and D1, but completely abolished the phosphorylation of LHCB1 and LHCB2 (Figure 3A). LHCII phosphorylation returned during the 1 h dark recovery, whereas the prolonged 24 h dark recovery led to almost complete dephosphorylation of the D1, D2 and LHCII proteins in both GT/GL control and LT/HL‐treated lettuce. In turn, the recovery in growth conditions restored thylakoid protein phosphorylation to the level of GT/GL control plants. Strikingly, we also detected strong phosphorylation of the minor antenna protein LHCB4 in the LT/HL‐treated lettuce (Figure 3A). This prompted us to analyse the effects of the LT/HL treatment and recovery on the organisation of the pigment‐binding thylakoid protein complexes, using CN gel electrophoresis (Figure 3B). After electrophoresis, the fluorescence of the separated complexes was analysed to reveal the possible involvement of qH (Bru et al. 2022), as a component of the strong sNPQ, but no major changes in the fluorescence of L‐LHCII trimers were detected (Figure 3C). On the other hand, the CN gels showed reduced amounts of PSII sc and increased amounts of M‐LHCII in LT/HL‐treated lettuce compared to the GT/GL control (Figure 3B). Detached M‐LHCII was not fully reassociated with the PSII core during dark recovery, as the amount of PSII sc remained at a lower level than in GT/GL controls, which could be caused by degradation of damaged PSII cores or degradation of M‐LHCII. These results were consistent with the fluorescence analysis of the gels (Figure 3C), which also showed a reduction in the fluorescence of monomeric PSII complexes in thylakoids isolated from LT/HL‐treated lettuce, which was also visible after 1 h of dark recovery.

To confirm the identity of the LT/HL‐induced phosphoprotein as p‐LHCB4 (Figure 3A), we separated the thylakoid proteins by 2D gel electrophoresis and visualised them with a phosphoprotein‐specific stain (Figure 3D). The position of the p‐LHCB4 in the 2D gels was affected by the choice of detergent in the native gel electrophoresis used for separation in the first dimension. With β‐dodecyl maltoside (β‐DM) as a solubilising agent, the phosphoprotein was found to migrate between L‐LHCII trimers and monomeric antenna proteins (Figure 3D, red arrow), whereas with digitonin solubilisation, it migrated in the M‐LHCII complex (Figure 3E, red arrow). M‐LHCII is composed of the LHCB1/LHCB3 trimer together with the LHCB4 and LHCB6 proteins, confirming the identification of the additional phosphoprotein as p‐LHCB4, the only component of M‐LHCII with the same mobility in CN. This suggested that the association of p‐LHCB4 with M‐LHCII is relatively weak, as it is detached from the complex with β‐DM (Figure 3D, red arrow), which is a slightly stronger detergent than digitonin. Furthermore, p‐LHCB4 was not detected in PSII sc (Figure 3D,E), further suggesting that LHCB4 phosphorylation detaches M‐LHCII from the PSII core.

Further information on changes in the relative antenna sizes of PSI and PSII during LT/HL treatment and subsequent recovery was obtained from the 77 K Chl fluorescence emission spectra recorded from isolated thylakoids. The LT/HL treatment increased the fluorescence emission ratio of PSI to PSII (F735/F685) from 1.11 in GT/GL control to 1.61 (Figure 3F), which is consistent with the detachment of M‐LHCII from PSII sc, thereby reducing the relative excitation of PSII. Recovery in darkness for 1 h further increased the fluorescence ratio to 1.72 compared to 1.02 of the GT/GL control, suggesting that the increased phosphorylation of LHCB2, which is associated with the LHCII‐PSI complex formation, has an additional effect on top of the detachment of M‐LHCII from PSII sc. After 24 h recovery in the dark, the thylakoids of LT/HL‐treated lettuce still showed a higher F735/F685 ratio (1.31) than that of the GT/GL controls (1.03). This could be due to the still reduced amount of PSII sc in the 24 h recovered plants compared to the GT/GL control (Figure 3B). The increased F735/F685 ratio after LT/HL treatment persisted not only after the 24 h dark recovery but also after recovery in light compared to the GT/GL control (Figure 3B).

### 3.3 Changes in Abundance of Distinct Thylakoid Proteins During LT/HL Treatment and Subsequent Recovery

To gain a more comprehensive insight into the mechanisms involved in the quenching of excitation energy in LT/HL‐treated lettuce and during the subsequent sNPQ relaxation, we took a targeted proteomic approach to gain a broader view on how the proteins of PSII core, light harvesting system and repair cycle were affected by the experiment. To this end, proteins from isolated thylakoids were detergent‐solubilised, digested with trypsin, and the resulting peptides were analysed by nLC‐ESI‐FAIMS‐MS/MS in DIA mode, which provides reliable protein quantification.

#### 3.3.1 LT/HL Treatment Leads to Only Minor Depletion of the PSII Core Proteins

**FIGURE 4:**
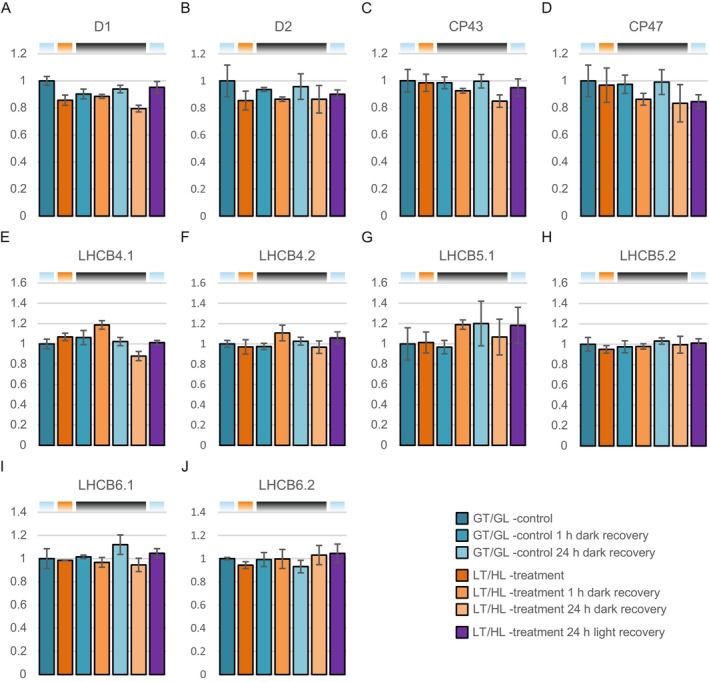
Effect of the low temperature and high light treatment, and the subsequent recovery, on major PSII core complex proteins and minor antenna proteins. (A) D1. (B) D2. (C) CP43. (D) CP47. (E) LHCB4.1. (F) LHCB4.2. (G) LHCB5.1. (H) LHCB5.2. (I) LHCB6.1. (J) LHCB6.2. Long day‐grown lettuce plants were illuminated under 1500 μmol photons m^−2^ s^−1^ of white light at 13°C for 4 h (LT/HL), while control plants were kept at growth conditions (23°C and 140 μmol photons m^−2^ s^−1^ with 16 h photoperiod) (GT/GL), after which all plants were transferred to recover for 1 and 24 h in darkness or for 24 h in long day growth conditions. Thylakoid membranes used in the analyses were isolated directly after the treatment and the recovery periods. Isolated thylakoids were solubilised with detergent and isolated proteins were digested with trypsin. Resulted peptide mixtures were analysed with nLC‐ESI‐FAIMS‐MS/MS in DIA mode and protein abundances were determined with Spectronaut software. Protein abundances were normalised to the average of control plants in growth light. Error bars show standard deviations among technical replicates (*n* = 3). Coloured bars above the graphs represent the temperature and light conditions from which the thylakoids used in the analyses were isolated: Light blue for GT/GL, orange for LT/HL and black for GT/darkness.

PSII photoinhibition damages D1 and D2 proteins and induces qI_RC_. Therefore, we analysed the levels of major PSII core proteins and minor antenna proteins (Figure 4). The decrease in the levels of the PSII RC proteins D1 and D2 was small, about 15%–20%, and occurred during the LT/HL treatment and remained at the reduced level also during the subsequent recovery in darkness (Figure 4A,B). Notably, the levels of the inner antenna proteins CP47 and CP43 remained stable during the LT/HL treatment, but a small fraction of them was degraded during recovery in darkness and light (Figure 4C,D), significantly later than for the PSII RC proteins D1 and D2. The amounts of the minor light‐harvesting antenna (LHCB4‐6) were slightly variable (Figure 4E–J), but there were no trends like those observed for the PSII core proteins.

#### 3.3.2 A Specific Subset of PSII Repair Machinery Is Up‐Regulated by LT/HL Treatment

**FIGURE 5:**
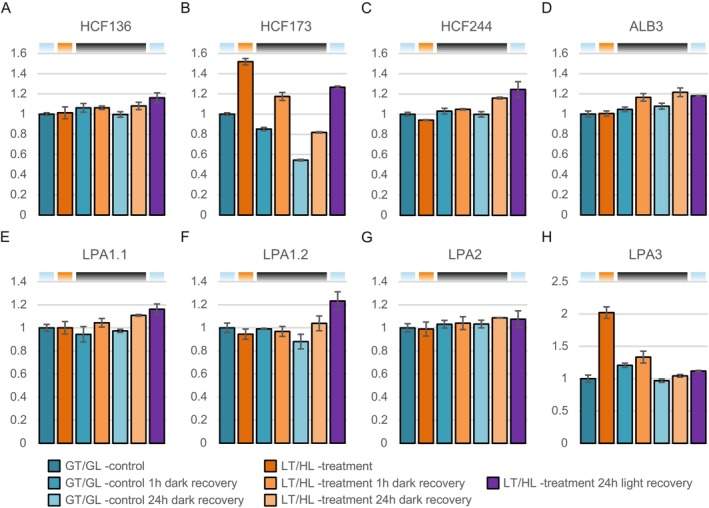
Effect of low temperature and high light treatment, and the subsequent recovery, on PSII repair and assembly factors. (A) High chlorophyll fluorescence 136 (HCF136). (B) High chlorophyll fluorescence 173 (HCF173). (C) High chlorophyll fluorescence 244 (HCF244). (D) Albino 3 (ALB3). (E) Low PSII accumulation 1.1 (LPA1.1). (F) Low PSII accumulation 1.2 (LPA1.2). (G) Low PSII accumulation 2 (LPA2). (H) Low PSII accumulation 3. (LPA3). Long day‐grown lettuce plants were illuminated under 1500 μmol photons m^−2^ s^−1^ of white light at 13°C for 4 h (LT/HL), while control plants were kept at growth conditions (23°C and 140 μmol photons m^−2^ s^−1^ with 16 h photoperiod) (GT/GL), after which all plants were transferred to recover for 1 and 24 h in darkness or for 24 h in long day growth conditions. Thylakoid membranes used in the analyses were isolated from leaves directly after the treatment and the recovery periods. Isolated thylakoids were solubilised with detergent and isolated proteins were digested with trypsin. Resulted peptide mixtures were analysed with nLC‐ESI‐FAIMS‐MS/MS in DIA mode and protein abundances were determined with Spectronaut software. Protein abundances were normalised to the average of control plants in growth light. Error bars show standard deviations among technical replicates (*n* = 3). Coloured bars above the graphs represent the temperature and light conditions from which the thylakoids used in the analyses were isolated: Light blue for GT/GL, orange for LT/HL and black for GT/darkness.

Since we detected changes in the fluorescence of PSII monomers (Figure 3C), associated with the PSII repair cycle, we focused on proteins known to be involved in PSII repair and biogenesis, such as high chlorophyll fluorescence (HCF) 173, HCF244, albino 3 (ALB3) and low PSII accumulation proteins (LPA1, LPA2 and LPA3) (Figure 5). HCF173 binds psbA transcripts and assists in translation initiation, and the interaction between HCF173 and HCF244 recruits D1‐translating polysomes to the thylakoid membrane (Schult et al. 2007; Link et al. 2012; Chotewutmontri and Barkan 2020; Wang and Grimm 2021). ALB3 is required for the insertion of newly translated D1 into the CP43‐less PSII (Schneider et al. 2014), whereas HCF136 (Ycf48 in cyanobacteria) has a functional role in preventing the premature formation of the oxygen‐evolving complex (Zhao et al. 2023). In addition, LPA1 has been implicated in the initial stages of D1 insertion, and it acts in concert with HCF136, whereas LPA2 and LPA3 have been reported to act in later stages of PSII assembly and repair by binding free CP43 and supporting its rebinding to the RC47 PSII intermediate (Nickelsen and Rengstl 2013; Schneider et al. 2014).

The abundance of HCF136, HCF244 and ALB3 increased slightly during the dark recovery of LT/HL‐treated plants compared to the control (Figure 5A,C,D), but in the case of HCF136 and HCF244, the clearly highest increase was observed in plants recovered in the light, where PSII repair is active (Figure 5A,C). HCF173 behaved differently and increased in abundance already during the LT/HL treatment (Figure 5B). During the dark recovery, the abundance of HCF173 decreased in both LT/HL‐treated and GT/GL control lettuce, but it remained at a higher level in LT/HL‐treated lettuce than in the control lettuce (Figure 5B). Among the LPA proteins, only the amount of LPA3 increased during the LT/HL treatment and then decreased to the control levels during the dark recovery (Figure 5E–H). These results suggest that HCF173 and LPA3 have specific functions during the LT/HL treatment and subsequent recovery.

#### 3.3.3 Specific LIL Proteins Show Differential Accumulation During LT/HL Treatment and Subsequent Recovery

**FIGURE 6:**
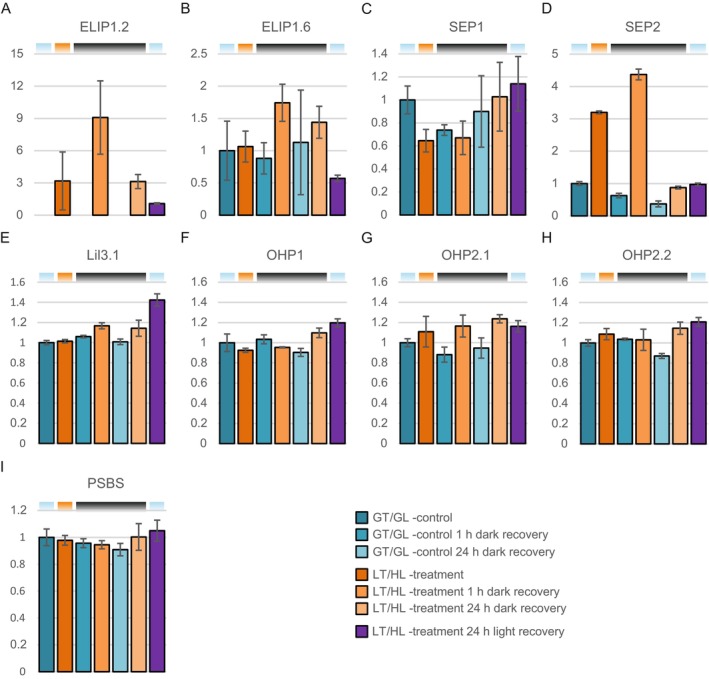
Effect of low temperature and high light treatment, and subsequent recovery, on light‐harvesting‐like proteins. (A) Early light‐induced protein 1.2 (ELIP1.2, normalised to plants recovered for 24 h in growth conditions). (B) Early light‐induced protein 1.6 (ELIP1.6). (C) Stress‐enhanced protein 1 (SEP1). (D) Stress‐enhanced protein 2 (SEP2). (E) Light‐harvesting‐like 3:1 (LIL3.1). (F) One helix protein 1 (OHP1). (G) One helix protein 2.1 (OHP2.1). (H) One helix protein 2.2 (OHP2.2). (I) Photosystem II subunit S (PSBS). Long day‐grown lettuce plants were illuminated under 1500 μmol photons m^−2^ s^−1^ of white light at 13°C for 4 h (LT/HL), while control plants were kept at growth conditions (23°C and 140 μmol photons m^−2^ s^−1^ with 16 h photoperiod) (GT/GL), after which all plants were transferred to recover for 1 and 24 h in darkness or for 24 h in long day growth conditions. Thylakoid membranes used in the analyses were isolated directly after the treatment and the recovery periods. Isolated thylakoids were solubilised with detergent and isolated proteins were digested with trypsin. Resulted peptide mixtures were analysed with nLC‐ESI‐FAIMS‐MS/MS in DIA mode and protein abundances were determined with Spectronaut software. Protein abundances were normalised to the average of control plants in growth light. Error bars show standard deviations among technical replicates (*n* = 3). Coloured bars above the graphs represent the temperature and light conditions from which the thylakoids used in the analyses were isolated: Light blue for GT/GL, orange for LT/HL and black for GT/darkness.

LIL proteins have been associated with sNPQ in previous studies (Demmig‐Adams et al. 2006; Zarter, Adams, Ebbert, Adamska, et al. 2006; Zarter, Adams, Ebbert, Cuthbertson, et al. 2006). Therefore, the accumulation of this protein family was checked after the LT/HL treatment and subsequent dark recovery (Figure 6). The lettuce genome contains several *LIL1* genes encoding the early light‐induced proteins (ELIP) (File S7). Of these seven isoforms, we detected only two, designated ELIP1.2 and ELIP1.6 (Figure 6A,B), in LT/HL‐treated and recovered samples. Accumulation of ELIP1.2 was induced by the LT/HL treatment and its abundance continued to increase together with ELIP1.6 during 1 h recovery in darkness. Recovery for 24 h in darkness reduced the levels of ELIPs, particularly ELIP1.2, similar to those after the LT/HL treatment, whereas recovery for 24 h in growth conditions returned the levels to control or even lower levels. ELIP1.2 was not detected in control samples, probably due to its low levels in unstressed plants and, for this reason, the amount of ELIP1.2 was normalised to that measured in plants that had recovered for 24 h under growth conditions.

Surprisingly, the two stress‐enhanced proteins SEP1 and SEP2 (Heddad and Adamska 2000), encoded by the *LIL4* and *LIL5* genes, behaved differently from each other (Figure 6C,D). The amount of SEP1 (quantified on a single peptide basis) decreased during the LT/HL treatment, whereas the amount of SEP2 increased during this treatment and especially during the 1 h recovery in darkness. In contrast to ELIPs, SEP2 levels returned to control levels during the 24 h recovery in darkness, suggesting that these stress‐related proteins may have different functions. The levels of other LIL proteins, light‐harvesting‐like 3:1 (LIL3.1), one helix protein 1 (OHP1), one helix protein 2.1 (OHP2.1) and one helix protein 2.2 (OHP2.2), encoded by the *LIL3.1*, *LIL2*, *LIL6.1* and *LIL6.2* genes, accumulated mainly in plants that had recovered from LT/HL treatment in the light (Figure 6E–H). OHPs, which are involved in D1 translation in a complex with HCF244 (Hey and Grimm 2018), showed similar trends of accumulation with HCF244 during the LT/HL treatment and subsequent recovery (Figures 5C and 6F–H). Lettuce has two isoforms for LIL3 (File S6), but we could only detect LIL3.1. Finally, the abundance of another LIL protein, PSBS, which is required for fast qE induction, did not change throughout the experiment (Figure 6I). Of all the LIL proteins detected, only the abundance of SEP2, together with ELIP1.2, correlated with LT/HL‐induced sNPQ formation and relaxation.

#### 3.3.4 LT/HL Treatment and Subsequent Recovery Alter the Amounts of Proteins Involved in the Metabolism of Photosynthetic Pigments

**FIGURE 7:**
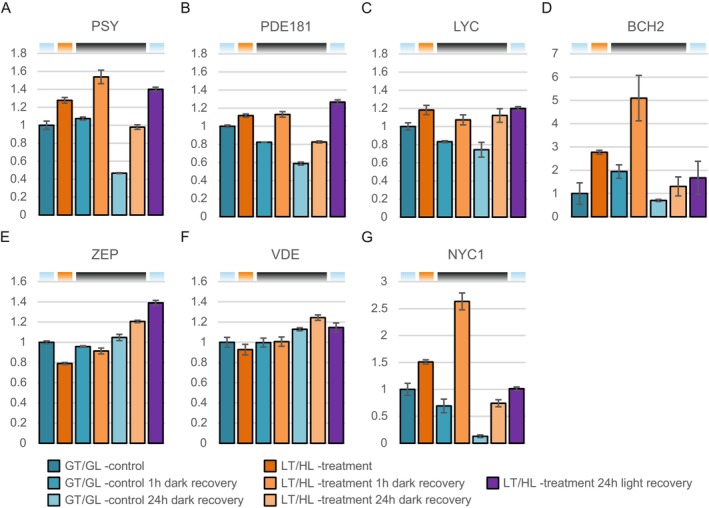
Effect of low temperature and high light treatment, and the subsequent recovery, on enzymes in the metabolism of photosynthetic pigments. (A) Phytoene synthase (PSY). (B) ζ‐carotene desaturase (PDE181). (C) Lycopene β‐cyclase (LYC). (D) β‐carotene hydroxylase (BCH2). (E) Zeaxanthin epoxidase (ZEP). (F) Violaxanthin de‐epoxidase (VDE). (G) Chlorophyll b reductase (NYC1). Long day‐grown lettuce plants were illuminated under 1500 μmol photons m^−2^ s^−1^ of white light at 13°C for 4 h (LT/HL), while control plants were kept at growth conditions (23°C and 140 μmol photons m^−2^ s^−1^ with 16 h photoperiod) (GT/GL), after which all plants were transferred to recover for 1 and 24 h in darkness or for 24 h in long day growth conditions. Thylakoid membranes used in the analyses were isolated directly after plant treatment and the recovery periods. Isolated thylakoids were solubilised with detergent and isolated proteins were digested with trypsin. Resulted peptide mixtures were analysed with nLC‐ESI‐FAIMS‐MS/MS in DIA mode and protein abundances were determined with Spectronaut software. Protein abundances were normalised to the average of control plants in growth light. Error bars show standard deviations among technical replicates (*n* = 3). Coloured bars above the graphs represent the temperature and light conditions from which the thylakoids used in the analyses were isolated: Light blue for GT/GL, orange for LT/HL and black for GT/darkness.

Since zeaxanthin accumulation is an important part of qZ, we focused on enzymes involved in pigment biosynthesis and the xanthophyll cycle (Figure 7). The LT/HL treatment resulted in a large increase in the amount of β‐carotene 3‐hydroxylase (BCH2) (Figure 7D), which synthesises zeaxanthin from β‐carotene. BCH2 further increased during the 1 h recovery in darkness before returning to control levels after the 24 h recovery in darkness or growth conditions. BCH2 is up‐regulated by β‐ionone, an oxidation product of β‐carotene (Felemban et al. 2023), suggesting that β‐carotene oxidation induces zeaxanthin synthesis. In addition, three enzymes leading to β‐carotene formation, the 15‐cis phytoene synthase (PSY), zeta‐carotene desaturase (PDE181) and lycopene β‐cyclase (LYC), had slightly higher abundances after LT/HL treatment when compared to GT/GL control, and the amounts did not decrease as much during the dark recovery as in the controls (Figure 7A–C). The levels of the xanthophyll cycle enzymes, zeaxanthin epoxidase (ZEP) and violaxanthin de‐epoxidase (VDE), were not induced; ZEP was actually decreased by LT/HL treatment, while an increase in ZEP and VDE was observed after a 24 h recovery period (Figure 7E,F). ZEP levels have been shown to decrease during HL treatment (Bethmann et al. 2019), but in addition, we detected an increasing trend in ZEP and VDE levels during dark and light recovery after the LT/HL treatment. Notably, the LT/HL treatment increased substantially the amount of Chl *b* reductase (NYC1), which reached the highest level after 1 h dark recovery compared to GT/GL controls (Figure 7G). NYC1 catalyses the first step of Chl *b* degradation, which is associated with the degradation of LHCII under HL illumination (Sato et al. 2015).

#### 3.3.5 LT/HL Treatment and Subsequent Recovery Have Little Effect on the Abundance of Enzymes Involved in Thylakoid Protein Phosphorylation or Proteins Required for qH Induction and Relaxation

To gain more information about the regulation of photosynthesis in our experimental conditions, we analysed the proteins involved in thylakoid protein phosphorylation and qH (Figure S2). State Transition 7 (STN7) phosphorylates LHCII, which is required for the formation of the LHCII‐PSI complex, whereas State Transition 8 (STN8) phosphorylates the PSII core proteins CP43, D1, D2, PSBH and the minor antenna protein LHCB4. LT/HL treatment had little effect on the abundance of the two kinases, but a moderate increase occurred during the 24 h recovery period for both STN8 and STN7 (Figure S2A,B). The levels of Thylakoid‐Associated Phosphatase 38 (TAP38) and Photosystem II Core Phosphatase (PBCP) decreased during the LT/HL treatment, and both recovered to GT/GL control levels during dark recovery (Figure S2C,D). Levels of STN8, TAP38 and PBCP were highest in plants that had recovered for 24 h under growth conditions.

The amount of Chloroplastic Lipocalin (CHL), which is required for qH formation in LHCII trimers, was not altered by the LT/HL treatment or the subsequent recovery (Figure S2G). The amount of Suppressor of Quenching 1 (SOQ1), which inhibits CHL, decreased slightly during the LT/HL treatment but was restored during recovery, where it even increased to a higher level than in control (Figure S2E). The amount of relaxation of qH (ROQH), which discharges the quenching at qH active LHCII, showed more changes in abundance. The abundance decreased slightly during the LT/HL treatment, and even more during dark recovery; however, in this case, the decrease occurred both in GT/GL control and LT/HL‐treated plants, suggesting that in lettuce the putative qH relaxation is not related to the LT/HL treatment and PSII recovery from photoinhibition (Figure S2F).

#### 3.3.6 LT/HL Treatment and Subsequent Recovery Induce Phosphorylation Dynamics of the PSII Core Proteins and the Minor LHCII Antenna Protein LHCB4.1

**FIGURE 8:**
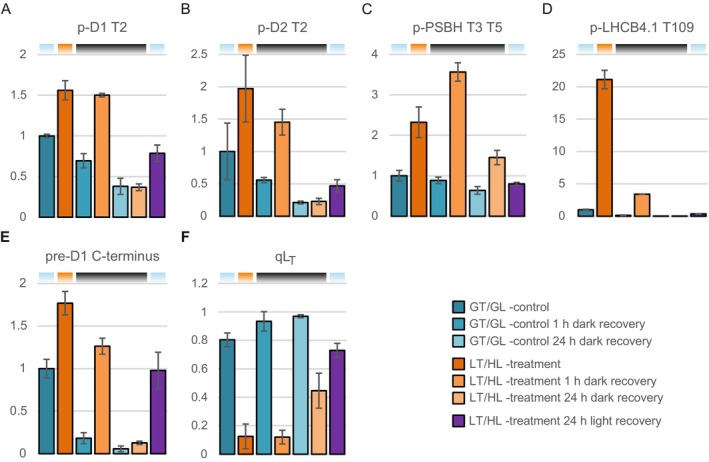
Effect of low temperature and high light treatment, and the subsequent recovery of lettuce plants, on phosphorylation of the PSII core proteins and the minor antenna LHCB4, D1 processing and recovery of PSII function. (A) p‐D1 T2. (B) p‐D2 T2. (C) 2‐pPSBH T3 T5. (D) p‐LHCB4.1 T109. (E) pre‐D1 C‐terminus. (F) Fraction of open and functional PSII reaction centre. Long day‐grown lettuce plants were illuminated under 1500 μmol photons m^−2^ s^−1^ of white light at 13°C for 4 h (LT/HL), while control plants were kept at growth conditions (23°C and 140 μmol photons m^−2^ s^−1^ with 16 h photoperiod) (GT/GL), after which all plants were transferred to recover for 1 and 24 h in darkness or for 24 h in long day growth conditions. Thylakoid membranes used in the analyses were isolated directly after the treatment and the recovery periods. Isolated thylakoids were solubilised with detergent and isolated proteins were digested with trypsin. Resulted peptide mixtures were analysed with nLC‐ESI‐FAIMS‐MS/MS in DIA mode and peptide abundances were determined with Spectronaut software. Peptide abundances were normalised to the average of control plants in growth light. Specific peptides for p‐D1 T2, p‐D2 T2, 2p‐PSBH T3 T5, p‐LHCB4.1 T109 and pre D1 are [N‐acetyl]T[p]AILER, [N‐acetyl]T[p]IALGKVTK, AT[p]QT[p]VENGAR, NLAGDVIGT[p]RFEDADVK and NAHNFPLDLAAIEAPSTNG respectively. *F*_0_′, *F*_0_, *F*_*M*_′, and *F*_*M*_ were determined with Fluorpen directly after the treatment and after subsequent recovery periods of 1 and 24 h. *qL*_*T*_ was calculated as ((1/*F*_0_′)−(1/*F*_*M*_′))/((1/*F*_0_^ref^)−(1/*F*_*M*_^ref^)) using as the reference values (*F*_*M*_^ref^ and *F*_0_^ref^) the averages of *F*_*M*_ and *F*_0_ from control plants shifted to darkness for 24 h. Error bars show standard deviations among technical replicates (A–E) (*n* = 3) and biological replicates (F) (*n* = 4–16). Coloured bars above the graphs represent the temperature and light conditions from which the thylakoids used in the analyses were isolated: Light blue for GT/GL, orange for LT/HL and black for GT/darkness.

Following the detection of LHCB4 phosphorylation by p‐Thr antibody and phosphoprotein staining of 2D gels (Figure 3A,E), the LHCB4 phosphorylation was further investigated by quantifying p‐LHCB4.1 and identifying the phosphosite from our MS data. In parallel to LHCB4.1, we also examined the phosphorylation dynamics of the PSII core proteins D1, D2 and PSBH (Figure 8A–C), which are known targets of the STN8 kinase. Since the phosphorylation and dephosphorylation of the D1 and D2 proteins are important parts of the PSII repair cycle, it was of interest to analyse the phosphorylation dynamics of these proteins. LT/HL treatment induced N‐terminal D1 and D2 phosphorylation, which remained high after 1 h dark recovery but decreased within 24 h dark recovery (Figure 8A,B). PSBH, a small PSII subunit with a transmembrane helix and a long stromal extension located at the interface of CP47 and LHCB4 (Su et al. 2017), was also phosphorylated during the LT/HL treatment, but curiously, the phosphorylation of T3 and T5 increased even further during 1 h dark recovery until it returned closer to the control levels after 24 h dark recovery (Figure 8C). The effect of LT/HL treatment on LHCB4.1 T109 phosphorylation was even more dramatic, being 20‐fold higher in LT/HL‐treated plants than control plants, but rapidly returning to control levels during 1 h dark recovery (Figure 8D). LHCB4.1 T109 phosphorylation site is located in the stromal loop of LHCB4.1, which in the PSII‐LHCII complex is bound to CP47 on the stromal side of the complex (Figure S3). Therefore, the phosphorylation of T109 could affect the interaction between LHCB4.1 and CP47, possibly explaining the detachment of M‐LHCII from the PSII core (Figure 3B,C).

#### 3.3.7 LT/HL Treatment Leads to the Accumulation of Pre‐D1 Protein, Which Allows Partial Restoration of PSII Function During Dark Recovery

More detailed analysis of the C‐terminal pre‐D1 peptide revealed a delay during the LT/HL treatment in the processing of the C‐terminal extension of the D1 protein by CTPA (Figure 8E), which is required for the assembly of functional PSII complexes (Che et al. 2013), indicating slow D1 turnover and PSII repair during the LT/HL treatment. Conversely, the pre‐D1 levels decreased during subsequent dark recovery both in control and LT/HL‐treated plants, implying that D1 protein C‐terminal processing, as well as some later steps of PSII repair, can proceed in the dark (Pavlovič et al. 2016). The cleavage of the D1 C‐terminal extension by CTPA allows the assembly of photochemically functional PSII (Che et al. 2013).

Since the PSII quantum yield, Y(II), also increased during dark recovery of LT/HL‐treated plants (Figure 2A), we next calculated the fraction of open and functional PSII centres (*qL*_*T*_) (Porcar‐Castell 2011) in control and LT/HL‐treated plants (Figure 8F). *qL*_*T*_ can be used to assess the amount of functional PSII centres since the PQ pool is mostly oxidised in darkness. *qL*_*T*_ was low, 0.13, directly after the LT/HL treatment and subsequent 1 h recovery in the dark, but increased to 0.44 during 24 h in dark recovery. These results strongly suggest that directly after the LT/HL treatment, a great portion of the mature D1 was damaged in photoinhibited PSII RCs, and the recovery in *qL*_*T*_ resulted from resumed processing of the D1 C‐terminal extension by CTPA.

### 3.4 LT/HL Treatment and Subsequent Recovery Alter the Pigment Composition of Lettuce Thylakoids

**FIGURE 9:**
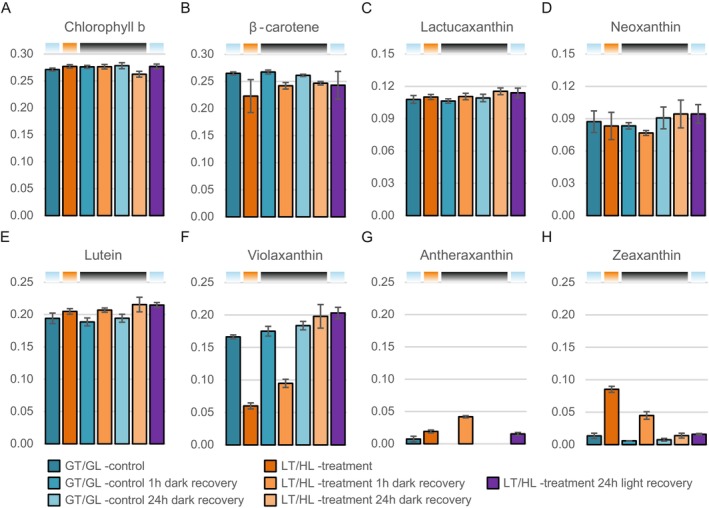
Effect of low temperature and high light treatment, and the subsequent recovery, on photosynthetic pigments. (A) Chlorophyll *b*. (B) β‐carotene. (C) Lactucaxanthin. (D) Neoxanthin. (E) Lutein. (F) Violaxanthin. (G) Antheraxanthin. (H) Zeaxanthin. Long day‐grown lettuce plants were illuminated under 1500 μmol photons m^−2^ s^−1^ of white light at 13°C for 4 h (LT/HL), while control plants were kept at growth conditions (23°C and 140 μmol photons m^−2^ s^−1^ with 16 h photoperiod) (GT/GL), after which all plants were transferred to recover for 1 and 24 h in darkness or for 24 h in long day growth conditions. Thylakoid membranes used in the analyses were isolated directly after the treatment and the recovery periods. Pigments were extracted from isolated thylakoids with acetone and the extracts were analysed with HPLC‐DAD. Pigments were identified by absorbance spectra and relative retention times. Relative pigment abundances were estimated by the area of chromatographic peaks detected at 440 nm and normalised within the sample to the area of Chl *a*. Error bars show standard deviations among four biological replicates (*n* = 4). Coloured bars above the graphs represent the temperature and light conditions from which the thylakoids used in the analyses were isolated: Light blue for GT/GL, orange for LT/HL and black for GT/darkness.

Since our proteomic analysis suggested an increase in Chl *b* catabolism and zeaxanthin synthesis after the LT/HL treatment and especially after 1 h dark recovery (Figure 7), this prompted us to analyse the pigment composition of isolated thylakoids. However, the decrease in the relative amount of Chl *b* was very small and only visible after 24 h of dark recovery (Figure 9A). In contrast, the decrease in β‐carotene, mainly bound to the PSI and PSII cores, occurred already during the LT/HL treatment (Figure 9B), which could be related to minor degradation of PSII core proteins and hydroxylation of released β‐carotene to zeaxanthin (Figure 4A–D) (Beisel et al. 2010). LT/HL treatment and subsequent recovery also increased the levels of all xanthophylls, with lactucaxanthin and neoxanthin being less affected (Figure 9C–H), consistent with previous studies (Esteban et al. 2015) and our proteomic analysis (Figure 7).

## 4 Discussion

### 4.1 Canonical NPQ Mechanisms Do Not Play a Major Role in LT/HL‐Induced sNPQ in Lettuce

qE is the fastest NPQ component induced in the light and depends on lumen acidification followed by protonation of the PSBS protein, with the strength of qE proportional to the expression level of the PSBS protein (Li et al. 2002). Because qE is dependent on lumen acidification, it relaxes within seconds in the dark (Niyogi and Truong 2013). In our lettuce experiments, we used 1 h as the shortest dark recovery time, which allowed about 70% relaxation of the NPQ induced by GT/HL treatment, but only about 30% relaxation of the NPQ induced by LT/HL treatment (Figure 2B). The amount of PSBS remained stable throughout the LT/HL treatment and subsequent recovery periods (Figure 6I), suggesting that the magnitude of qE during LT/HL treatment is similar to that during the GT/HL treatment, and appears to be fully relaxed within 1 h dark recovery (Figure 2B).

Slightly slower relaxing NPQ mechanisms in plants, compared to qE, have been attributed to qZ, which is based on the retention of zeaxanthin in the external LHCII antenna (Nilkens et al. 2010). Zeaxanthin is formed in the light‐induced xanthophyll cycle under abiotic stress conditions (Demmig‐Adams and Adams 2006) and promotes the quenching of excitation energy in the external LHCII antenna systems (Bassi and Dall'Osto 2021; Ruban and Saccon 2022). By the end of the 1 h dark recovery, about half of the zeaxanthin had been converted to antheraxanthin (Figure 9G,H), which is unlikely to contribute to qZ because zeaxanthin de‐epoxidation releases the formed antheraxanthin from LHCII into the lipid bilayer (Küster et al. 2023). Therefore, the remaining amount of zeaxanthin can only account for part of the high sNPQ remaining after 1 h dark recovery of LT/HL‐treated lettuce (Figures 2B and 9H). Furthermore, zeaxanthin accumulation does not correlate with sNPQ in all plant species (Míguez et al. 2015, 2017). Taken together, these results suggest that the LT/HL‐induced sNPQ, which is preserved after 1 h of dark recovery, does not represent the PSBS or zeaxanthin‐dependent NPQ, and therefore other sNPQ mechanisms must exist in lettuce.

We next considered a different form of sNPQ, a lipocalin‐dependent qH described in Arabidopsis (Malnoë 2018). However, its involvement in the sNPQ generated in lettuce during the LT/HL treatment and its subsequent relaxation during dark recovery is difficult to assess, due to the lack of clear molecular signatures to track qH. The known qH‐related proteins, CHL, SOQ1 and ROQH1, showed no differential expression in LT/HL‐treated lettuce compared to GT/GL control (Figure S2E–G). Slightly reduced fluorescence of L‐LHCII trimers in CN gels has been attributed to qH in Arabidopsis (Bru et al. 2022). In lettuce, however, no apparent changes in the fluorescence of L‐LHCII trimers were observed during LT/HL treatment or subsequent recovery phases (Figure 3C), suggesting that qH is not particularly active under the stress conditions applied here to lettuce.

### 4.2 Role of LHCB4 Phosphorylation in the Formation of sNPQ in LT/HL‐Treated Lettuce?

Canonical LHCII phosphorylation‐dependent quenching (qT) is not expected to occur under HL in plants, because the STN7 kinase, which phosphorylates LHCB1 and LHCB2, is inhibited under such conditions, preventing the formation of the LHCII‐PSI sc (Rintamäki et al. 2000; Bassi and Dall'Osto 2021; Cutolo et al. 2023). As expected, after the LT/HL treatment of lettuce, we detected almost complete dephosphorylation of LHCB1 and LHCB2. However, a major phosphorylation of LHCB4 appeared during the LT/HL treatment (Figure 3A), which in angiosperms has previously been reported only in grasses (Chen et al. 2013; Betterle et al. 2017). Grasses, lettuce, and most angiosperms have two isoforms of LHCB4 (LHCB4.1 and LHCB4.2), whereas Arabidopsis, pea ( *Pisum sativum* ) and spinach ( *Spinacia oleracea* ), which are commonly used in photosynthesis research, have three isoforms (LHCB4.1, LHCB4.2 and LHCB4.3), of which the LHCB4.3 is expressed only in HL, leading to dissociation of the M‐LHCII trimer and LHCB6 from the PSII core (Albanese et al. 2019; Grebe et al. 2019).

**FIGURE 10:**
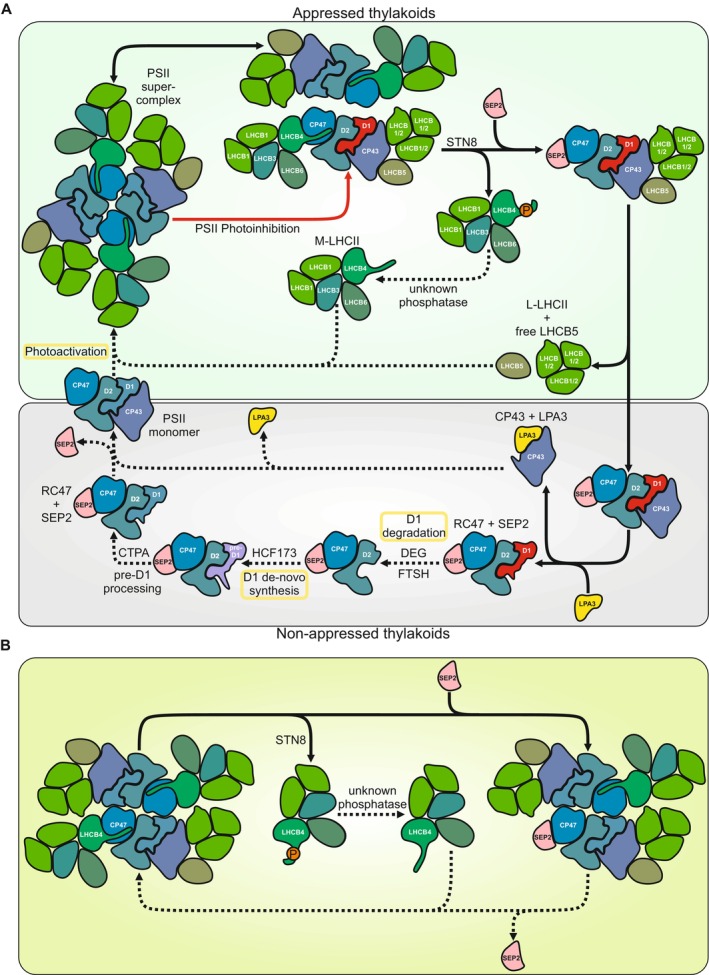
Proposed roles of STN8‐dependent LHCB4 phosphorylation and accumulation of SEP2 and LPA3 proteins upon stalling of PSII repair during the LT/HL treatment (A), and the role of STN8 in the formation of sNPQ in functional PSII complexes (B) in lettuce. Solid arrows represent processes occurring during the LT/HL treatment: STN8‐dependent phosphorylation of LHCB4 leads to the dissociation of M‐LHCII from the PSII sc. The release of M‐LHCII allows SEP2 to bind to CP47 and quench the excitation energy at the inner antenna of PSII, either in damaged CP47RC (A) or in functional PSII (B) cores. Release of CP43 from damaged PSII complexes, resulting in RC47 complexes, allows the degradation of the non‐phosphorylated damaged D1 protein. LPA3 binds to free CP43, protecting it from photodamage and degradation (A). Dashed arrows represent processes that are slowed down during the LT/HL treatment and resumed during subsequent recovery: Dephosphorylation of LHCB4 and degradation of SEP2 allow reassociation of M‐LHCII with PSII cores (A, B). The paused PSII repair cycle is completed by degradation of dephosphorylated damaged D1 and co‐translational insertion of a new D1 copy into the RC47 complex. CTPA catalysed pre‐D1 processing allows the re‐binding of CP43 and photoactivation of PSII (A). Processes marked with a yellow rectangle are light‐dependent and therefore do not occur during dark recovery. PSII photoinhibition and damaged D1 proteins are shown in red, but marking of phosphorylation of the PSII core proteins is omitted for clarity.

Interestingly, the phosphorylation of lettuce LHCB4 was found to occur on the stromal loop, at the site that serves as a binding site for CP47 in unphosphorylated LHCB4 (Figure S3). It is therefore conceivable that the STN8‐dependent phosphorylation of LHCB4 (Betterle et al. 2017) leads to the dissociation of p‐LHCB4, together with the attached M‐LHCII trimer and LHCB6 (i.e., the entire M‐LHCII complex), from the PSII core (Figures 3B and 10), performing the same function as LHCB4.3 in other species. This interpretation is consistent with the absence of p‐LHCB4 in PSII sc (Figure 3D,E). The dissociation of the M‐LHCII complex from the PSII core during the LT/HL treatment (Figure 3B) would decrease the relative antenna size of PSII, which is consistent with the increase in the F735/F685 ratio (Figure 3F). However, the increased F735/F685 ratio, partially disassembled PSII complexes, and high levels of free M‐LHCII were maintained after 1 h recovery in darkness, even though the LHCB4 proteins were already dephosphorylated (Figure 3A,B,F). This probably means that the reassociation of M‐LHCII with the PSII core is prevented by some other mechanism(s), as will be discussed in the following chapters. The reduction in PSII antenna size can explain part of the sNPQ, but based on the F735/F685 ratio, there must be also other mechanism to explain the high sNPQ.

### 4.3 Regulation of PSII Repair Leads to Pausing of D1 C‐Terminal Processing and Inhibition of D1 Degradation During LT/HL Treatment and Subsequent Dark Recovery, Leading to Accumulation of Different PSII Populations

Our analysis of PSII repair‐associated proteins revealed that LT/HL treatment induced a clear accumulation of HCF173 protein (Figure 5B). HCF173 binds *psbA* transcripts, recruits D1‐translating polysomes to the thylakoid membrane, and facilitates the insertion of nascent D1 chains into partially disassembled PSII subcomplexes (Schult et al. 2007; Link et al. 2012; Chotewutmontri and Barkan 2020; Wang and Grimm 2021). However, a clear pausing of D1 protein maturation during the PSII repair cycle under LT/HL was evident by the accumulation of the pre‐D1 protein (Figure 8E), which remained at a high level also after 1 h at recovery conditions in darkness and GT. This likely results from the inhibition of pre‐D1 C‐terminal processing protease (CTPA) by the thioredoxin system (Hall et al. 2010; Järvi et al. 2015), thus linking the regulation of PSII repair to stromal redox state. Only after 24 h of transfer of lettuce plants to recovery conditions in the dark, where no new initiations of D1 translation take place (Chotewutmontri and Barkan 2018), revealed the conversion of accumulated pre‐D1 to mature D1, apparently by activation of CTPA.

Not only the processing of pre‐D1, but also the degradation of damaged D1 proteins, was stalled during the LT/HL treatment and subsequent dark recovery, resulting in a great portion of the PSII centres with mature D1 protein being non‐functional, evidenced by a low *qL*_*T*_ (Figure 8F). Those PSIIs most likely represent the PSII complexes that, in order to gain functionality, need a replacement of the damaged D1 protein with a newly synthesised one. The accumulation of damaged D1 relates to the elevated N‐terminal phosphorylation of D1 protein (Figure 8A), which is known to prevent D1 degradation (Rintamä et al. 1996; Kato and Sakamoto 2014; Puthiyaveetil et al. 2014). Moreover, a slow dephosphorylation of D1 and D2 during the LT/HL treatment, in comparison to control plants (Figure 8A), implies that PBCP, the PSII core protein phosphatase, is inhibited by the LT/HL treatment (Liu et al. 2019). Thus, the degradation of damaged D1 is strictly regulated not only by kinases but also by phosphatases.

By comparison of the changes in the total D1 protein levels (Figure 4A) and in the amounts of pre‐D1 peptide (Figure 8E) with the *qL*_*T*_ values (Figure 8F), we estimate that during the LT/HL treatment, up to 20% of PSII RCs are degraded, approximately 40% of the PSII RCs have a damaged D1 protein, and 30% of the PSII RCs have a pre‐D1, while only 10% of the PSII RCs are functional (Figure S4). In comparison, the control plants without the LT/HL treatment comprised 80% active PSIIs with mature D1 protein, 15% with pre‐D1 and 5% with damaged D1 protein. Recovery in darkness for 24 h after the LT/HL treatment allowed the processing of the C‐terminal extension of the pre‐D1 protein by CTPA (Figure 8E), but the full recovery of PSII eventually requires also light (Figure 8F).

The accumulation of pre‐D1 during the LT/HL treatment coincides with the accumulation of the LPA3 protein (Figures 8E and 5H), which has been shown to interact with CP43 during the PSII repair cycle in Arabidopsis (Cai et al. 2010). D1 C‐terminal extension prevents the correct binding of CP43 to D1 during the repair cycle, leading to the accumulation of free CP43 (Che et al. 2013; Shi et al. 2021). The recently reported spectroscopic studies show that free CP43 isolated from Synechocystis (*Synechocystis* sp. PCC 6803) (Biswas et al. 2023) is poorly protected under excess illumination, leading the authors to speculate that some weakly interacting protein, lost during isolation, is likely to quench the free CP43 under more natural conditions. Considering these two reports, we propose that LPA3 binds to CP43 released from the PSII core upon initiation of the PSII repair cycle and protects the free CP43 from light‐induced damage and proteolytic degradation (Figures 4C, 5H and 10A).

### 4.4 SEP2‐Dependent Quenching of the PSII Inner Antenna CP47 as a Putative Mechanism for sNPQ in Lettuce

Among the LIL proteins in lettuce, the accumulation dynamics of the SEP2 protein coincided with the formation and relaxation of sNPQ (Figures 2B and 6D). Plant LIL proteins have evolved from cyanobacterial high light‐induced proteins (Hlip), which are replaced with the OHP and SEP proteins in the green lineage (Engelken et al. 2010). Synechocystis has four Hlips that are involved in PSII assembly and repair (Komenda and Sobotka 2016). The HliCD heterodimer delivers pigments to the newly translated D1 protein and protects the complex from light‐induced damage (Knoppová et al. 2014; Staleva et al. 2015). In plants, the OHP proteins are functional equivalents of Synechocystis HliC and HliD, but in lettuce, OHPs showed only minor changes in abundance during the LT/HL treatment or subsequent recovery (Figure 6F–H). On the other hand, Synechocystis HliA and HliB form heterodimers with HliC, and the dimers bind to free CP47 and to the PSII assembly intermediate RC47 complex (Konert et al. 2022). The HliAC heterodimer appears to be involved in PSII repair, whereas the HliBC heterodimer functions in the assembly of CP47 (Rahimzadeh‐Karvansara et al. 2022). Related to the different functions of HliA and HliB in Synechocystis during HL stress (Konert et al. 2022; Rahimzadeh‐Karvansara et al. 2022), we found similarities in the behaviour of the SEP1 and SEP2 proteins in lettuce under our experimental conditions (Figure 6C,D). While the amount of SEP1 decreased during the LT/HL treatment (Figure 6C), the SEP2 levels increased substantially and coincided with the apparent stalling of the PSII repair cycle. These evolutionary and functional considerations led us to hypothesise that lettuce SEP2 binds to PSII repair intermediates and quenches them (Figures 3C and 10A).

Further analysis of evolutionary aspects revealed that Synechocystis HliA and HliB interact also with the Psb35 protein, which is thought to protect CP47, HliA and HliB from FtsH‐mediated degradation by covering the N‐terminal parts of these proteins (Pascual‐Aznar et al. 2021). Lettuce and plants in general do not have Psb35, but the second helix of SEP1 shares some homology with Psb35 (Pascual‐Aznar et al. 2021). Moreover, Psb35 and the HliA and HliB proteins are fused in some Synechococcus strains (Kilian et al. 2008), suggesting that such an early evolutionary event could be linked to the evolution of SEPs and OHPs in the green lineage. Intriguingly, the HliA and HliB proteins have been proposed to bind to CP47 in Synechocystis at the same site where LHCB4 in plants binds to CP47 (Pascual‐Aznar et al. 2021). It is therefore conceivable that SEP2 binds to the same site in both damaged and functional PSII complexes. In the latter case, the binding of SEP2 to CP47 would explain why LHCB4 in lettuce, after dephosphorylation, does not rebind to the PSII core during 1 h recovery in darkness (Figures 3A,C and 8D). The binding of SEP2 to CP47 could promote energy dissipation as heat rather than directing light to the PSII RC. Such a competition between the binding of SEP2 and LHCB4 to the PSII core would provide an additional regulatory loop between light harvesting and photoprotection in lettuce (Figure 10B).

### 4.5 Putative Role of ELIP1.2 Accumulation During the LT/HL Treatment and Subsequent 1 h Recovery in Darkness

ELIPs, a distinct group of LIL proteins, have been proposed to function as pigment carriers (Adamska 1997; Tzvetkova‐Chevolleau et al. 2007). In lettuce, ELIP1.2 accumulation occurred during the LT/HL treatment but even more prominently during the subsequent 1 h recovery in the dark (Figure 6A), coinciding with the degradation of the damaged PSII cores (Figure 4A–D), leading us to suggest that Chls released from damaged PSII cores are bound to ELIP1.2 under conditions where the PSII repair cycle is not fully active (Figure S5) (Beisel et al. 2010). A similar coincidence between the accumulation of damaged PSII cores and the accumulation of ELIPs has been reported for overwintering evergreens (Zarter, Adams, Ebbert, Adamska, et al. 2006; Zarter, Adams, Ebbert, Cuthbertson, et al. 2006). The LT/HL treatment of lettuce also induced the synthesis of zeaxanthin, most likely by BCH2 catalysed hydrolysis of the β‐carotene released from the degradation of damaged PSII RCs (Figures 3A–D, 7D and 9B,H), as the abundance of BCH2 increased already during the LT/HL treatment but especially during the 1 h recovery in the dark (Figure 7D). ELIPs are known to also bind zeaxanthin (Rossini et al. 2006; Skotnicová et al. 2021), which would allow quenching of the bound Chls. In summary, ELIP1.2 is proposed to act as a safe storage site for Chl *a* until it is reused for PSII repair or ultimately degraded.

In addition to binding Chl *a* from damaged PSII complexes, lettuce ELIP1.2 may also have another role in rescuing both Chl *a* and Chl *b* from degraded LHCII (Figure S5). This interpretation is based on the fact that the abundance of the NYC1, which catalyses the first step of converting Chl *b* to Chl *a*, was up‐regulated in our experiment, especially after 1 h of dark recovery (Figure 7G). On the other hand, the accumulation of ELIP2 in Arabidopsis under constitutive expression has been shown to downregulate Chl biosynthesis (Tzvetkova‐Chevolleau et al. 2007). This suggests that the formation of ELIP1.2‐dependent Chl stores may also regulate Chl metabolism by inhibiting Chl biosynthesis when there is a high level of stored Chl. This hypothesis of ELIPs as safe pigment stores would also imply that ELIPs do not play a role in the formation of sNPQ, which is consistent with unchanged Y(II) in the ELIP‐overexpressing Arabidopsis (Tzvetkova‐Chevolleau et al. 2007).

## 5 Conclusions

Lettuce plants form strong NPQ during the LT/HL treatment due to excessive excitation stress. Based on biochemical, biophysical and quantitative targeted proteome analyses, we propose that the STN8 kinase‐dependent phosphorylation of LHCB4, detected in grasses and lettuce among the land plants, initially dissociates the M‐LHCII complex from PSII sc (Figure 10). This reduces the excitation energy transfer to the PSII cores and simultaneously increases the excitation lifetime in the shared LHCII pool, which increases the probability of excitation energy quenching by the qE‐active LHCII trimers, the qZ‐active minor LHCII antenna (LHCB4, LHCB5 and LHCB6) as well as via the PSI complexes. At the same time, however, the LT/HL treatment induces an additional strong sNPQ in lettuce, which is largely retained after 1 h recovery in darkness, when canonical NPQ mechanisms are largely relaxed. This novel sNPQ is related to the accumulation of SEP2, which is proposed to bind and quench the CP47 inner antenna of PSII core (Figure 10) while free CP43, accumulating due to the stalling of pre‐D1 maturation, is protected by LPA3. At the same time, STN8 kinase‐dependent phosphorylation hampers the degradation of damaged D1, leading to the accumulation of non‐functional PSII centres. On the other hand, a strong accumulation of ELIP1.2 as an early event during the LT/HL treatment and subsequent 1 h recovery in the dark seems to be important to bind and store the Chls released from damaged and degraded pigment‐protein complexes (Figure S5), thereby preventing the generation of singlet oxygen by free Chls.

It is important to emphasise that a physiologically relevant understanding of the regulation of the photosynthetic apparatus requires disentangling the roles of multiple and often condition‐specific mechanisms acting simultaneously in a highly controlled manner. Nevertheless, redox regulation is a unifying feature of a number of such mechanisms. It is therefore conceivable that excessive excitation stress triggers in the photosynthetic apparatus a wave of redox activation or inactivation of different enzymes and signalling cascades, leading to photoprotection of the photosynthetic apparatus and acclimation to the new conditions over a longer time scale. The hypotheses presented here would be a starting point for further in‐depth studies on the regulation of photoprotective networks in plant chloroplasts and far beyond.

## Author Contributions

This study was designed by the cooperation of all authors. T.L. performed all the biochemical and biophysical experiments. D.M.‐P. and J.P.V. performed the proteomic experiments and data analysis, together with T.L. T.L. and E.‐M.A. drafted the manuscript, which was commented on and approved by all authors.

## Supporting information


**File S1.** Moderate temperature reduction changes high‐light acclimation strategy of lettuce plants.

**File S2.** Lettuce SwissProt sequences.

**File S3.** BLASTs from removed sequences.

**File S4.** Sequences removed from combined database.

**File S5.** CURATED_uniprot_Lettuce_28proteome‐2023.02.15&120_SwissProt2023.03.28.

**File S6.** Photosynthesis related proteins in lettuce (based on homology to Arabidopsis).

**File S7.** LHC and LIL proteins in lettuce (based on homology to Arabidopsis).

**Data S1.** ppl70298‐sup‐0008‐Supinfo.

## Data Availability

The proteomics raw data supporting the findings of this work are available in the PRIDE Archive database (PXD055190). More detailed information is available on reasonable request from the corresponding authors.
