# Supplementary material for: Moderate Temperature Reduction Changes the High‐Light Acclimation Strategy of Lettuce Plants
Source: Physiol Plant. 2025 Jun 2;177(3):e70298. doi: 10.1111/ppl.70298 (PMC12130749; doi:10.1111/ppl.70298)
Supplement: Supplementary file 7 — File S7. LHC and LIL proteins in lettuce (based on homology to Arabidopsis). [file PPL-177-e70298-s002.zip › File S7_LHC and LIL proteins in lettuce (based on homology to Arabidopsis)/Lettuca and Arabidopsis LHC and LIL aligments.pptx]

## Slide 1
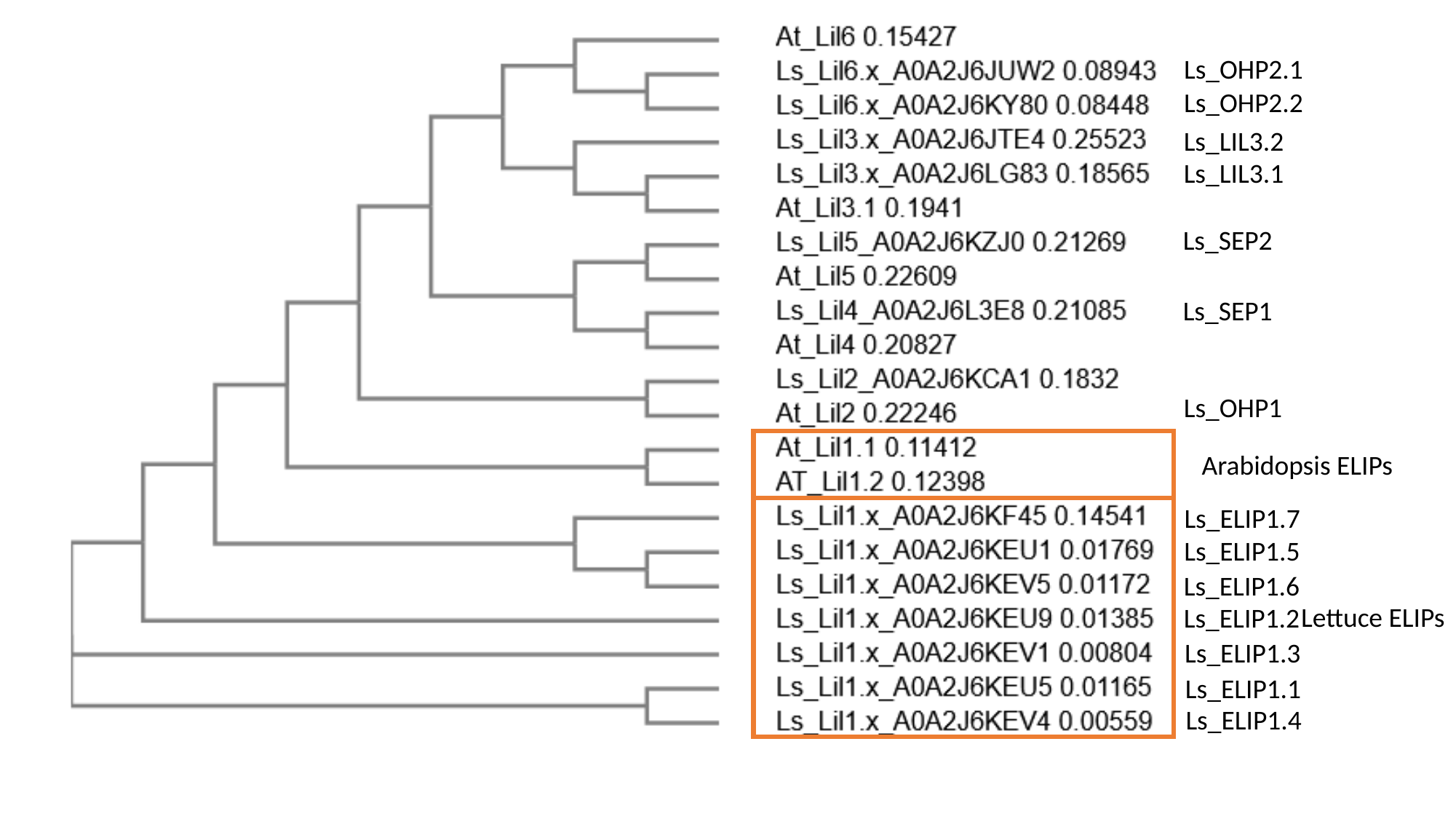

Ls_OHP2.1
Ls_OHP2.2
Ls_LIL3.2
Ls_LIL3.1
Ls_SEP2
Ls_SEP1
Ls_OHP1
Arabidopsis ELIPs
Ls_ELIP1.7
Ls_ELIP1.5
Ls_ELIP1.6
Lettuce ELIPs
Ls_ELIP1.2
Ls_ELIP1.3
Ls_ELIP1.1
Ls_ELIP1.4

## Slide 2
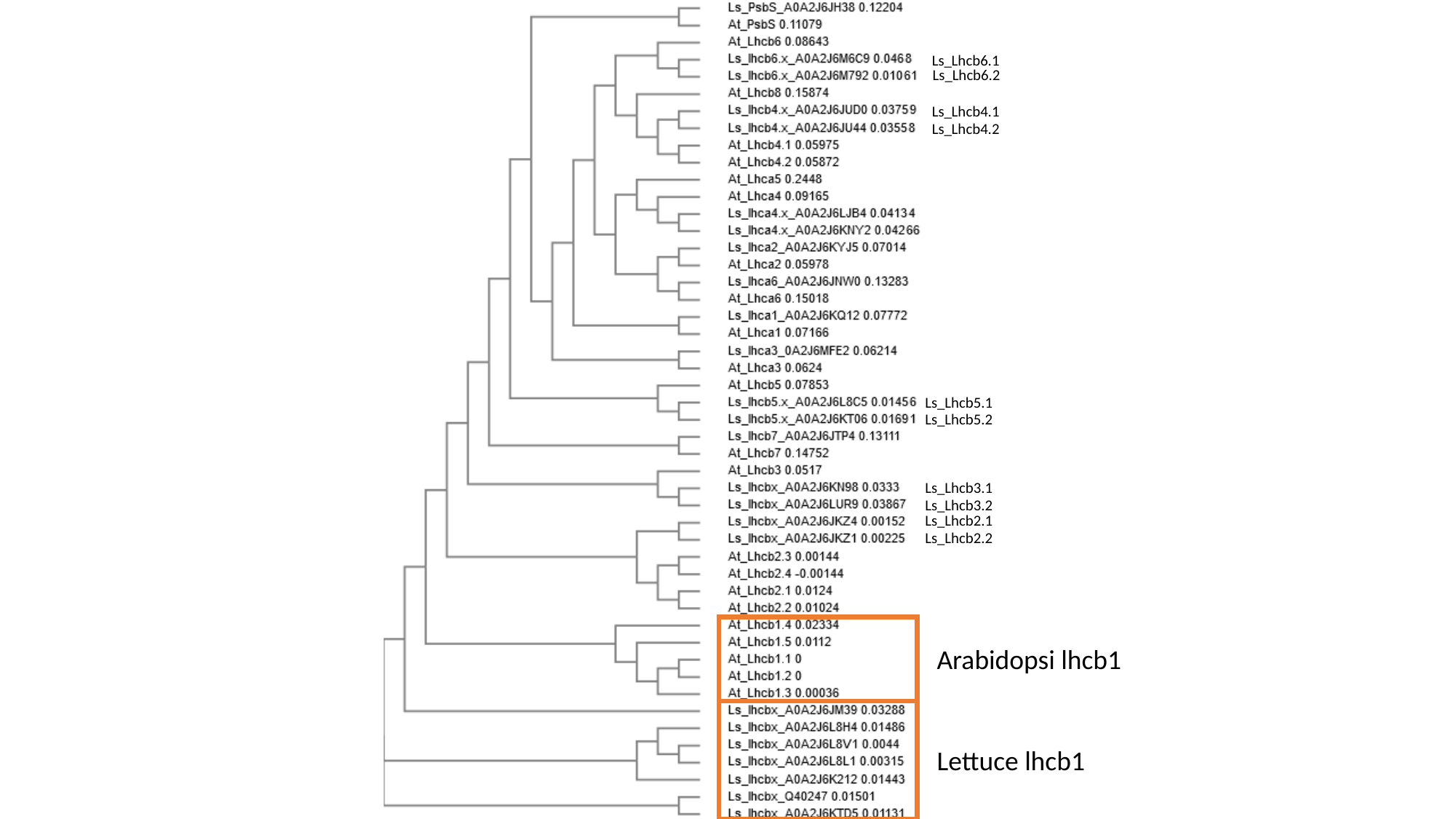

Ls_Lhcb6.1
Ls_Lhcb6.2
Ls_Lhcb4.1
Ls_Lhcb4.2
Ls_Lhcb5.1
Ls_Lhcb5.2
Ls_Lhcb3.1
Ls_Lhcb3.2
Ls_Lhcb2.1
Ls_Lhcb2.2
Arabidopsi lhcb1
Lettuce lhcb1
