# Supplementary figures and images for: Moderate Temperature Reduction Changes the High‐Light Acclimation Strategy of Lettuce Plants
Source: Physiol Plant. 2025 Jun 2;177(3):e70298. doi: 10.1111/ppl.70298 (PMC12130749; doi:10.1111/ppl.70298)

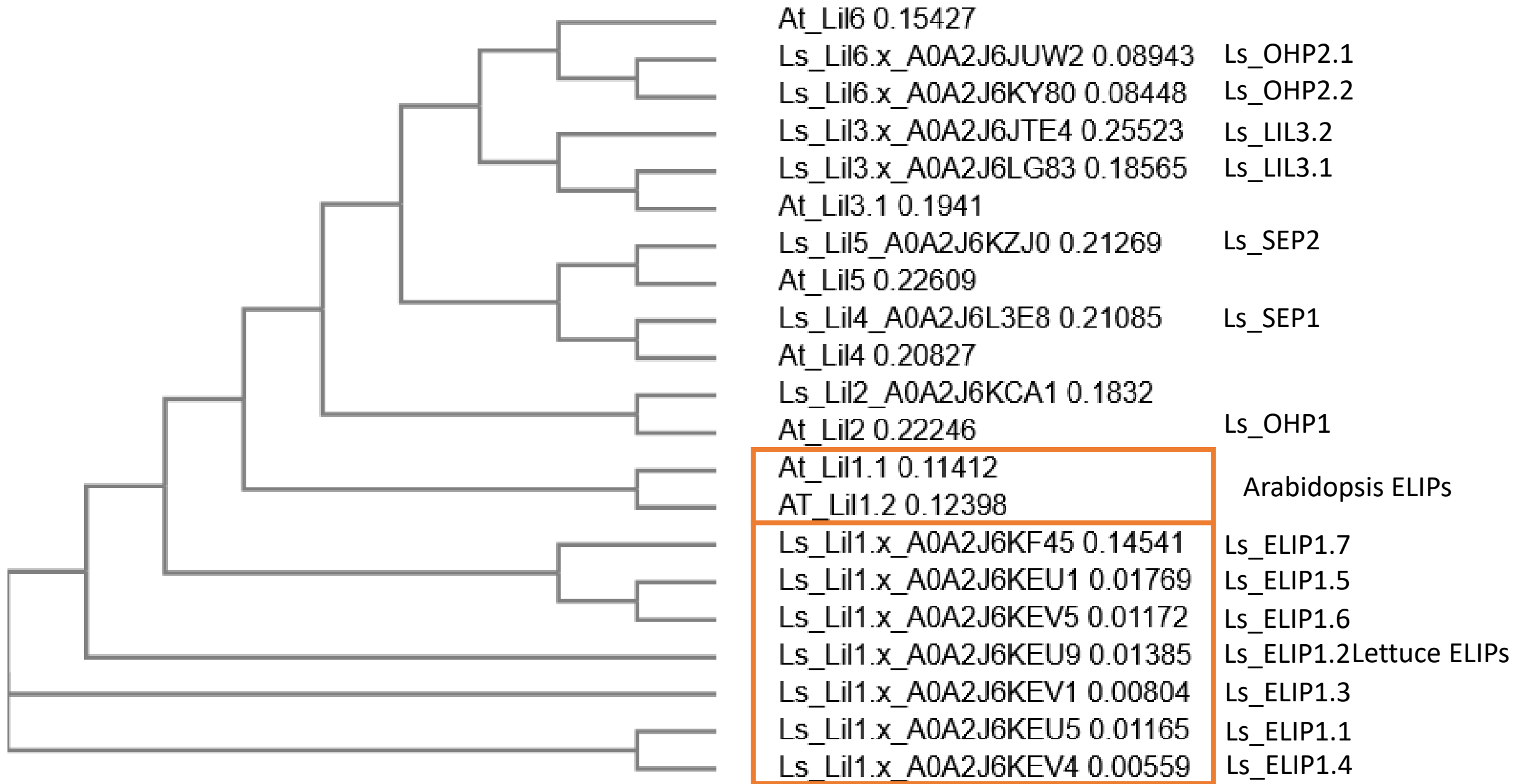

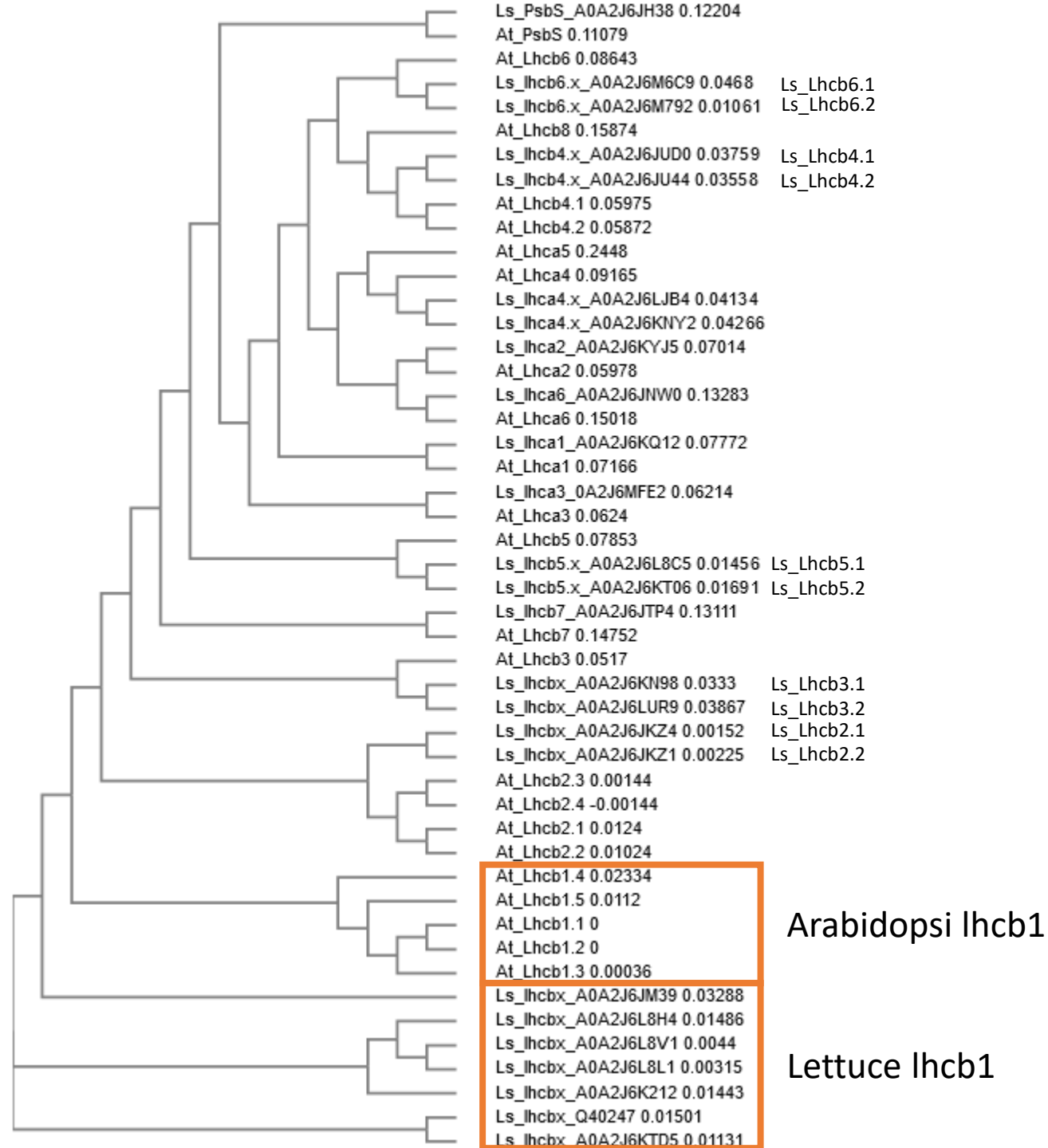

Supplement: Supplementary file 7 — File S7. LHC and LIL proteins in lettuce (based on homology to Arabidopsis). [file PPL-177-e70298-s002.zip › File S7_LHC and LIL proteins in lettuce (based on homology to Arabidopsis)/Lettuca and Arabidopsis LHC and LIL aligments.pdf]
