# Supplementary material for: Moderate Temperature Reduction Changes the High‐Light Acclimation Strategy of Lettuce Plants
Source: Physiol Plant. 2025 Jun 2;177(3):e70298. doi: 10.1111/ppl.70298 (PMC12130749; doi:10.1111/ppl.70298)
Supplement: Supplementary file 8 — Data S1. ppl70298‐sup‐0008‐Supinfo. [file PPL-177-e70298-s007.pdf]

Supplemental figures and Table

Moderate temperature reduction changes the high-light acclimation strategy of lettuce plants

Tapio Lempiäinen*, Dorota Muth-Pawlak, Julia P. Vainonen+, Eevi Rintamäki, Mikko Tikkanen,
Eva-Mari Aro*

Molecular Plant Biology, Department of Life Technologies, University of Turku, Turku, Finland
+ Current address: Organismal and Evolutionary Biology Research Programme, Faculty of Biological and Environmental Sciences, University of Helsinki, Helsinki, Finland
***Correspondence**
Tapio Lempiäinen: lempiainen.tapio@gmail.com,
Eva-Mari Aro evaaro@utu.fi

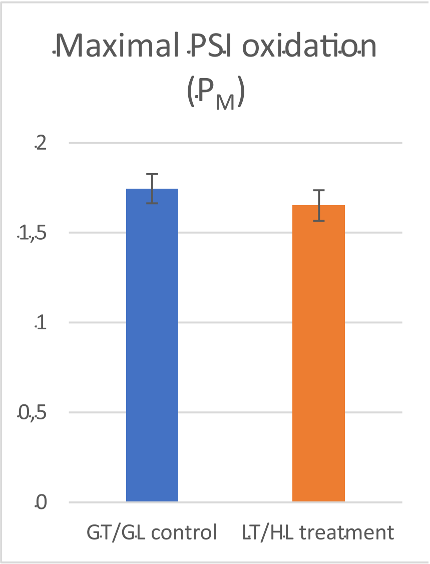

Figure S1. Effect of the 4h LT/HL treatment on maximal PSI oxidation (P_M). Long day-grown lettuce plants were illuminated under 1500 µmol photons m^{-2} s^{-1} of white light at 13 °C for 4 h (LT/HL). Control plants were kept at growth conditions (23 °C, 140 µmol photons m^{-2} s^{-1}) (GT/GL). P_M was determined with Dual KLAS-NIR after 1 h dark acclimation. Error bars show standard deviations among biological replicates (n = 6).

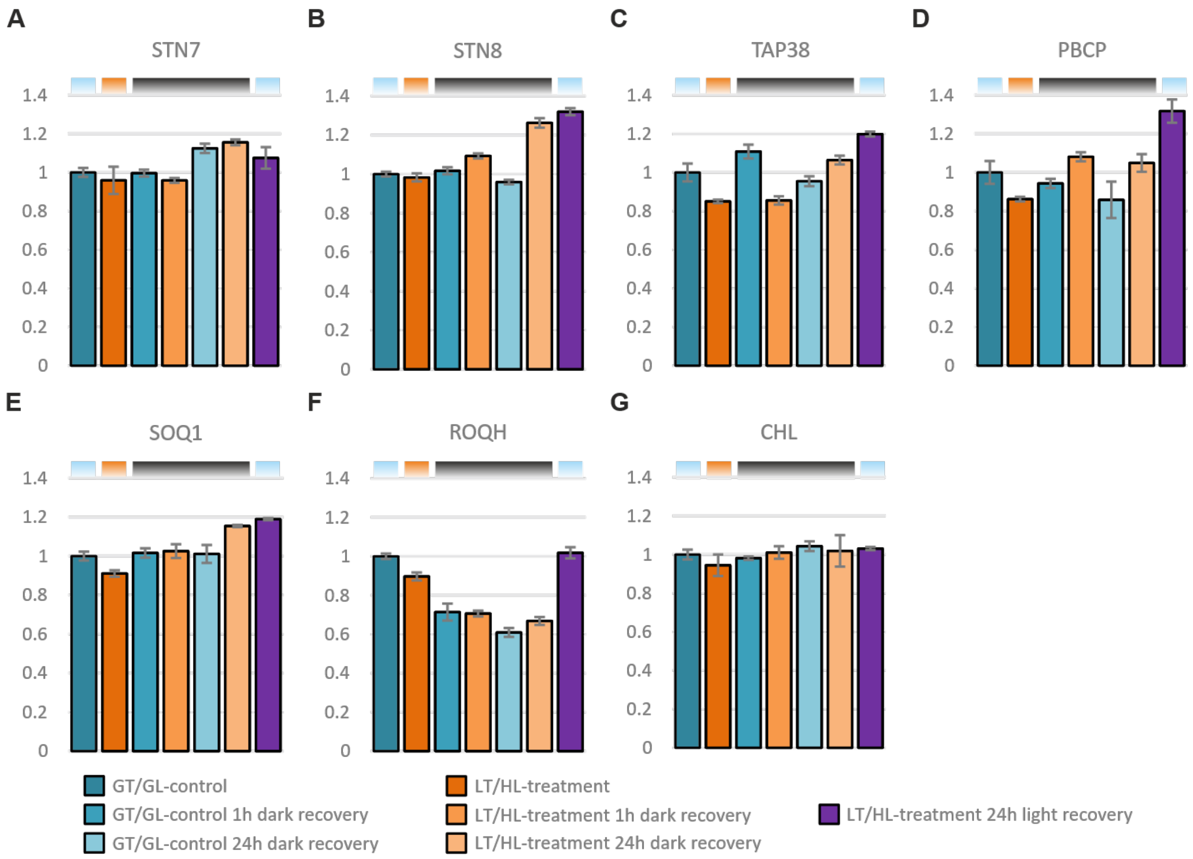

Figure S2. Effect of low temperature and high light treatment, and the subsequent recovery, on regulatory proteins of photosynthesis. **A)** State transition 7 (STN7) **B)** State transition 8 (STN8) **C)** Thylakoid-associated phosphatase 38 (TAP38) **D)** Photosystem II core phosphatase (PBCP) **E)** Suppressor of quenching 1 (SOQ1) **F)** Relaxation of qH (ROQH1) **G)** Chloroplastic lipocalin (CHL). Long day-grown lettuce plants were illuminated under 1500 µmol photons m−2 s−1 of white light at 13 °C for 4 h (LT/HL), while control plants were kept at growth conditions (23 °C and 140 µmol photons m−2 s−1 with 16 h photoperiod) (GT/GL), after which all plants were transferred to recover for 1 h and 24 h in darkness or for 24 h in long day growth conditions. Thylakoid membranes used in the analyses were isolated directly after the treatment and the recovery periods. Isolated thylakoids were solubilized with detergent and isolated proteins were digested with trypsin. Resulted peptide mixtures were analysed with nLC-ESI-FAIMS-MS/MS in DIA mode and protein abundances were determined with Spectronaut software. Protein abundances were normalised to the average of control plants in growth light. Error bars show standard deviations among technical replicates (n = 3). Coloured bars above the graphs represent the temperature and light conditions from which the thylakoids used in the analyses were isolated: light blue for GT/GL, orange for LT/HL and black for GT/darkness.

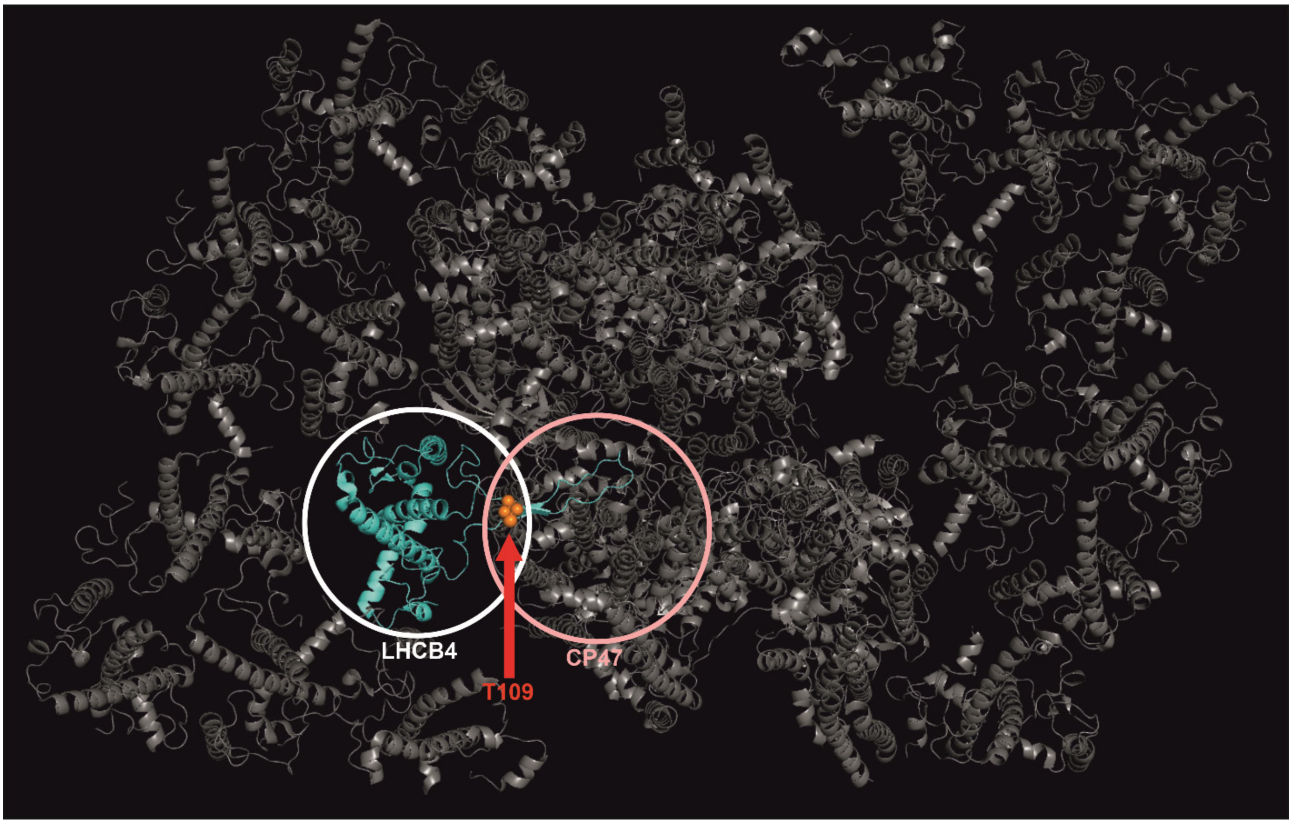

Figure S3. LHCB4 and CP47 interaction in the PSII sc and the localisation of T109, which is highly phosphorylated in lettuce treater for 4 h at low temperature and high light treated (1500 µmol photons m^{-2} s^{-1} of white light at 13 °C). The figure was generated using PyMol software and it is based on the structure of Arabidopsis sc (Su et al. 2017).

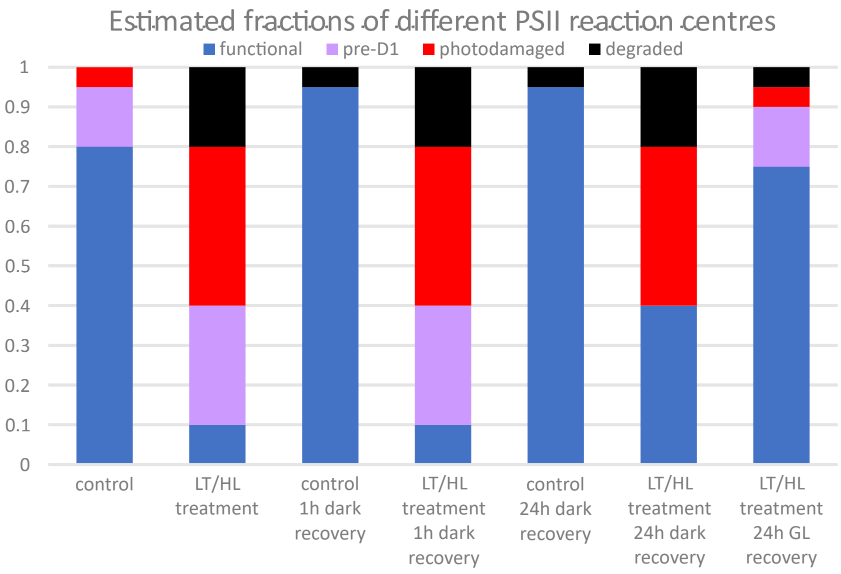

Figure S4. Estimation of different fractions of PSII during LT/HL-treatment and subsequent recovery.

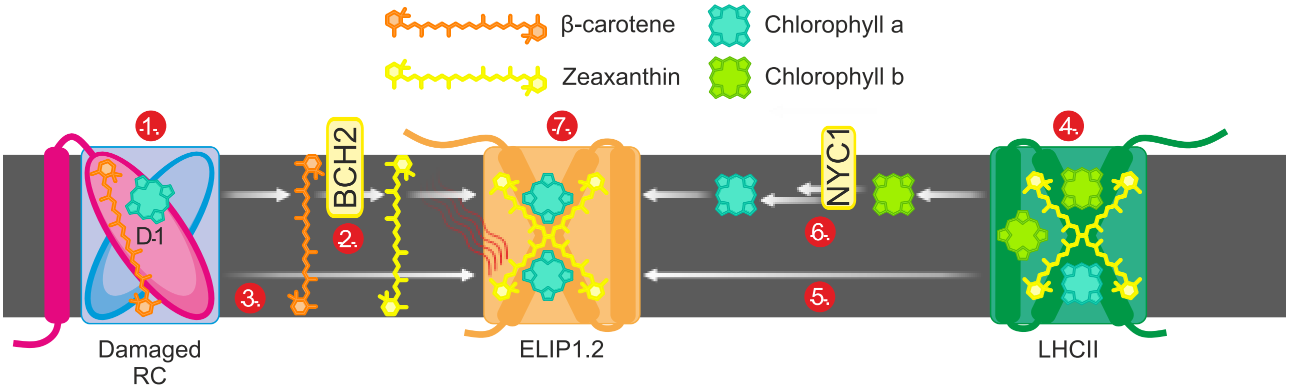

Figure S5. Proposed role for ELIP1.2 protein in storing the Chl from damaged and degraded PSII and LHCII in thylakoids of the LT/HL-treated lettuce: **1.** Damaged PSII cores are degraded by FTSH protease and pigments are released from the complex. **2.** The released β-carotene is hydrolysed by BCH2 (β-carotene hydroxylase) to zeaxanthin, which binds to the de-novo translated ELIP1.2. **3.** Chls released from degraded PSII cores are scavenged by ELIP1.2 when the Chl salvage pathway is not active enough to immediately incorporate the released Chl a into the PSII repair cycle. **4.** Degradation of LHCII releases bound Chl a and Chl b. **5.** Chl a released from LHCII is transferred to ELIP1.2. **6.** Reduction of Chl b to Chl a is initiated by NYC1 (chlorophyll b reductase) and the formed Chl a is bound to ELIP1.2. **7.** ELIP1.2 quenches the bound Chl a with zeaxanthin, making ELIP1.2 a safe pigment store until the bound Chl a can be reused or eventually degraded.

Table S1 List of tMS2 targeted masses (m/z) and the corresponding isolation windows (m/z) used in DIA measurements.

m/z	z	window width (m/z)
**415**	2	30
**442,5**	2	52
**464**	2	18
**480,5**	2	15
**495,5**	2	15
**510,5**	2	15
**525,5**	2	15
**540,5**	2	15
**555,5**	2	15
**570,5**	2	15
**585,5**	2	15
**600,5**	2	15
**615,5**	2	15
**630,5**	2	15
**645,5**	2	15
**660,5**	2	15
**676,5**	2	17
**697,5**	2	25
**722,5**	2	25

**750**	2	30
**780**	2	30
**817,5**	2	45
**865**	2	50
**945**	2	110
